# Safety assessment of basiliximab using real-world adverse event data from the FDA Adverse Event Reporting System Database: A retrospective observational study

**DOI:** 10.1097/MD.0000000000039537

**Published:** 2024-09-06

**Authors:** Sheng Chen, Xiaohan Ma, Jianqiang Zhang

**Affiliations:** a Department of Urology, The First People’s Hospital of Nanning, Nanning, Guangxi, China; b Graduate School, Guangxi University of Chinese Medicine, Nanning, Guangxi, China; c Ruikang Hospital, Guangxi University of Chinese Medicine, Nanning, Guangxi, China.

**Keywords:** adverse event, basiliximab, FAERS, infection, rejection

## Abstract

This study analyzed adverse drug events (ADEs) associated with basiliximab, sourced from the Food and Drug Administration Adverse Event Reporting System (FAERS) database, spanning the first quarter of 2004 to the fourth quarter of 2023. We collected ADE data for basiliximab from 2004 Q1 to 2023 Q4. After standardization, we employed several signal quantification methods for analysis, such as the Reporting Odds Ratio (ROR), Proportional Reporting Ratio (PRR), Bayesian Confidence Propensity for Neural Networks (BCPNN), and empirical bayes geometric mean (EBGM). In this analysis of 1520 ADEs reports citing basiliximab as the primary suspect, we identified 295 preferred terms across 24 system organ classifications (SOCs). The 3 most prevalent SOCs were investigated (n = 1403, ROR 2.84, PRR 2.54, IC 1.34, EBGM 2.54), infections and infestations (n = 1198, ROR 2.85, PRR 2.59, IC 1.37, EBGM 2.59), and renal and urinary disorders (n = 903, ROR 6.01, PRR 5.48, IC 2.45, EBGM 5.47). Increased blood creatinine and pyrexia were the most frequently reported adverse events (AEs) associated with basiliximab, and cytomegalovirus infection also demonstrated significant signal intensity. Notably, this study revealed some adverse reactions beyond basiliximab drug instructions, such as mitral valve calcification, diastolic dysfunction, pelvic fluid collection, testicular swelling, soft tissue necrosis, and muscle necrosis. Although basiliximab offers therapeutic benefits, it carries the risk of several adverse reactions. Clinicians should monitor patients for signs of increased serum creatinine level, fever, cytomegalovirus infection, anaphylactic shock, mitral valve calcification, diastolic dysfunction, pelvic fluid collection, testicular swelling, soft tissue necrosis, muscle necrosis, and other events during clinical use.

## 1. Introduction

Basiliximab, a chimeric monoclonal antibody, has been shown to effectively prevent early posttransplant kidney rejection, demonstrating significant advantages over placebo. Studies indicate that the use of basiliximab can enhance treatment outcomes without adversely affecting the 1-year survival rates of patients.^[1–4]^ This drug is particularly beneficial in individuals with renal impairment. Its use leads to a strategic delay in the administration of calcineurin inhibitors, which are known to potentially worsen kidney damage if introduced too early in the treatment process. By allowing this delay, basiliximab ensures a safer and more controlled treatment progression, minimizing the risks associated with premature intervention.^[5]^

Basiliximab, while generally effective in preventing transplant rejection, also presents potential side effects that necessitate thorough examination and vigilance.^[6]^ Numerous extensive literature reviews have methodically discussed these adverse effects and highlighted the importance of understanding their implications. However, issues such as publication bias in case series and individual reports may significantly distort the depiction of these reactions, leading to a potentially misleading understanding of drug safety.^[7–9]^ Consequently, this situation underscores the urgent need to develop and adopt more effective methods to systematically summarize and analyze these adverse reactions. Such an approach would ensure a more accurate and comprehensive understanding of the safety profile of basiliximab and contribute to better patient care and drug utilization strategies.

Pharmacovigilance plays a vital role in monitoring, evaluating, and effectively mitigating the adverse effects and issues associated with drug use, and is pivotal for ensuring patient safety and enhancing drug efficacy.^[10]^ In the modern healthcare landscape, pharmacovigilance databases have become indispensable tools for conducting real-world postmarketing studies. These databases are instrumental in ongoing safety assessments of medications currently in public use, providing essential data that help inform regulatory decisions and clinical practice.^[11]^ By systematically collecting and analyzing data on drug-related issues, these databases facilitate prompt identification and proactive addressing of any concerns, significantly improving healthcare outcomes by ensuring that medications remain safe and effective for patient populations.

The FAERS database plays a crucial role in the gathering and examination of drug-related adverse drug events (ADEs). This study employed FAERS data on basiliximab to meticulously analyze its safety through various signal quantification techniques.

## 2. Methods

### 2.1. Data source

This study utilized the American Standard Code for Information Interchange report files from the FAERS database.

### 2.2. Data extraction and analysis

**Figure 1.**
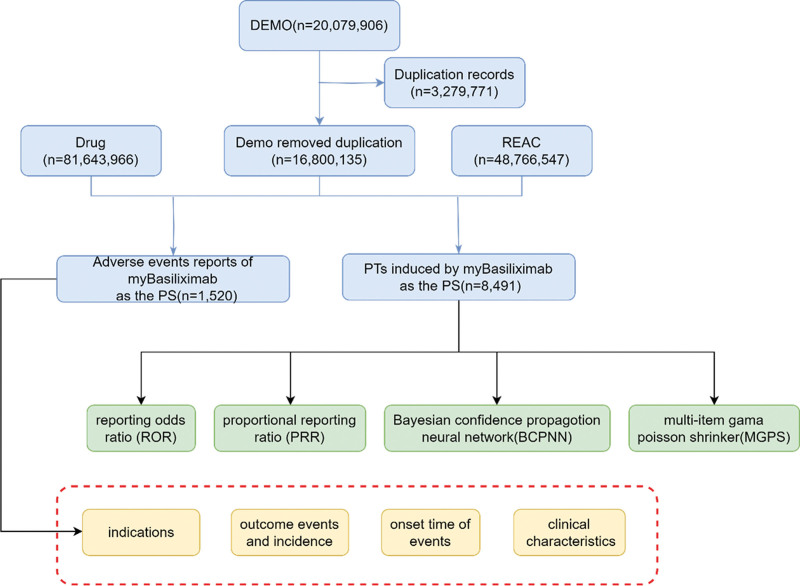
Flow diagram of selection of basiliximab-related AEs from the FAES database.

Redundant reports were removed, retaining only the most recent report for each unique case ID in the demographic database based on the date. Data were linked through the “primaryid” field, and reports identifying basiliximab as the primary drug associated with ADEs were selected. The study applied 4 disproportionality methods – Reporting Odds Ratio (ROR),^[12]^ Proportional Reporting Ratio (PRR),^[13]^Bayesian Confidence Propensity for Neural Networks (BCPNN),^[14]^ and empirical bayes geometric mean (EBGM)^[15]^ – were used to detect drug ADE signals. ROR addresses the bias from limited reports on specific events, PRR offers specificity, BCPNN excels in integrating and cross-validating data from various sources, and EBGM can detect signals from rare events. This combined approach leverages the strengths of each algorithm to broaden the detection scope, validate the findings from multiple perspectives, and ensure more reliable safety signal identification. Cross-validation across algorithms helps to reduce false positives. Adjustments in thresholds and variance enabled the identification of rare adverse reactions. All methods utilize 2 × 2 contingency tables, as detailed in Table 1, with precise calculations and threshold values in Table 2. Statistical analysis was performed using R. Higher values indicated stronger signal intensity, suggesting a more significant association between the drug and adverse events (AEs). Figure 1 illustrates the precise method used to identify AEs associated with basiliximab in the FAERS database.

**Table 1:** Four grid table.

	Basiliximab-related ADEs	Non-basiliximab-related ADEs	Total
Basiliximab	a	b	a + b
Non-basiliximab	c	d	c + d
Total	a + c	b + d	N = a + b + c + d

ADEs = adverse drug events.

**Table 2:** ROR, PRR, BCPNN, and EBGM methods, formulas, and thresholds.

Algorithms	Equation	Criteria
ROR	ROR = ad/b/c	lower limit of 95% CI > 1, N ≥ 3
	95%CI = e^ln(ROR)±1.96(1/a+1/b+1/c+1/d)^0.5^	
PRR	PRR = a(c + d)/c/(a + b)	PRR ≥ 2, χ^2^ ≥ 4, N ≥ 3
	χ^2^=[(ad-bc)^2](a + b + c + d)/[(a + b)(c + d)(a + c)(b + d)]	
BCPNN	IC = log_2_a(a + b + c + d)(a + c)(a + b)	IC025 > 0
	95%CI = E(IC) ± 2V(IC)^0.5	
MGPS	EBGM = a(a + b + c + d)/(a + c)/(a + b)	EBGM05 > 2
	95%CI = e^ln(EBGM)±1.96(1/a+1/b+1/c+1/d)^0.5^	

95%CI = 95% confidence interval, E(IC) = the IC expectations, EBGM = empirical Bayesian geometric mean, EBGM05 = the lower limit of 95% CI of EBGM, IC = information component, IC025 = the lower limit of 95% CI of the IC, MGPS = multi-item gamma Poisson shrinker, N = the number of reports, V(IC) = = the variance of IC, χ2 = chi-squared.

### 2.3. Signal filtering and categorization

The initial screening filtered for preferred terms (PTs) was reported 3 or more times. Using the Medical Dictionary for Regulatory Activities for PT and SOC, this study encoded and categorized signals for analysis, particularly focusing on system organ classifications (SOCs) related to AE signals.

## 3. Results

### 3.1. Basic characteristics of basiliximab-related AE reports

**Figure 2.**
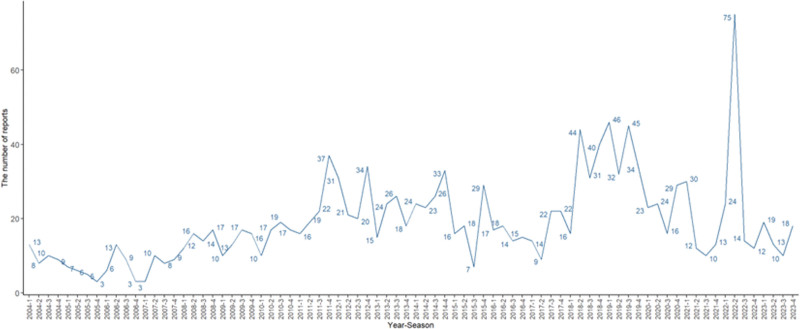
The number of AEs was reported quarterly after basiliximab marketing.

This study extracted 20,079,906 AE reports from the FAERS database from the first quarter of 2004 to the fourth quarter of 2023. Of these, basiliximab was highlighted as the first suspect in 1520 ADEs reports (Fig. 2). These AE reports were very unevenly distributed between females and males, with only 471 reports in females and 864 in males. A major challenge in this analysis was the lack of gender information in 12.17% of the reports, which limited a comprehensive study of the relationship between gender and AEs. Where age data were available, the 18 to 65 years age group dominated, but 21.58% of the reports lacked gender information, which is a major challenge in analyzing a comprehensive study of the age-AE relationship.

**Figure 3.**
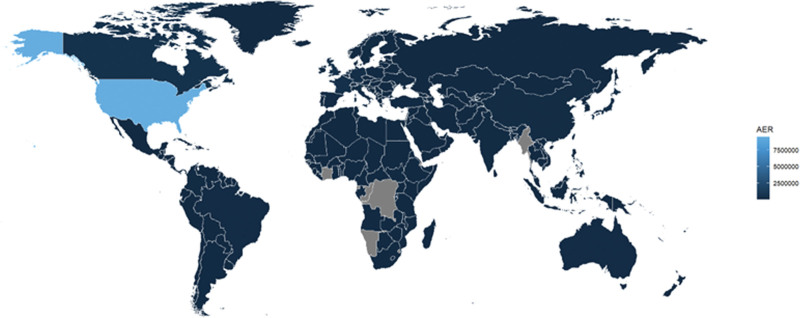
World distribution of adverse reactions.

The source of reporting was mainly other health professionals (39.41%) and consumers (14.28%), but physicians accounted for only 28.49%, which shows the lack of physicians reporting AEs. For Reported countries, the highest number of reports was from India (10.92%); details can be found in Figure 3. In terms of known types of clinical outcomes, hospitalization is the most common AE (31.18%), followed by death (13.93%). Further details are provided in Table 3.

**Table 3:** Basic information on ADEs related to basiliximab from the FAERS database.

Variable	Number of events (%)
Year	
2004	40 (2.63)
2005	21 (1.38)
2006	31 (2.04)
2007	30 (1.97)
2008	59 (3.88)
2009	56 (3.68)
2010	63 (4.14)
2011	94 (6.18)
2012	106 (6.97)
2013	83 (5.46)
2014	106 (6.97)
2015	70 (4.61)
2016	64 (4.21)
2017	67 (4.41)
2018	131 (8.62)
2019	157 (10.33)
2020	92 (6.05)
2021	65 (4.28)
2022	125 (8.22)
2023	60 (3.95)
Sex	
Female	471 (30.99)
Male	864 (56.84)
Unkown	185 (12.17)
Age	
<18	119 (7.83)
18 to 45	456 (30.00)
45 to 65	485 (31.91)
65 to 75	123 (8.09)
≥75	9 (0.59)
Unknown	328 (21.58)
Reporter	
Other health-professional	599 (39.41)
Physician	433 (28.49)
Consumer	217 (14.28)
Pharmacist	213 (14.01)
Unkown	56 (3.68)
Lawyer	1 (0.07)
Registered nurse	1 (0.07)
Reported countries	
Other	908 (59.74)
India	166 (10.92)
United States	163 (10.72)
China	116 (7.63)
Japan	110 (7.24)
France	57 (3.75)
Route	
Other	911 (59.93)
Intravenous	609 (40.07)
Outcomes	
Other serious	1033 (48.29)
Hospitalization	667 (31.18)
Death	298 (13.93)
Life threatening	112 (5.24)
Disability	26 (1.22)
Congenital anomaly	2 (0.09)
Required intervention to Prevent Permanent Impairment/Damage	1 (0.05)
Adverse event occurrence time - medication date (d)	
<7	310 (43.30)
7 to 28	129 (18.02)
28 to 60	63 (8.80)
≥60	97 (13.55)
Unknown	117 (16.34)

ADEs = adverse drug events, FAERS = Food and Drug Administration Adverse Event Reporting System.

### 3.2. Basiliximab signal mining

#### 3.2.1. Signals detection based on SOC levels

**Figure 4.**
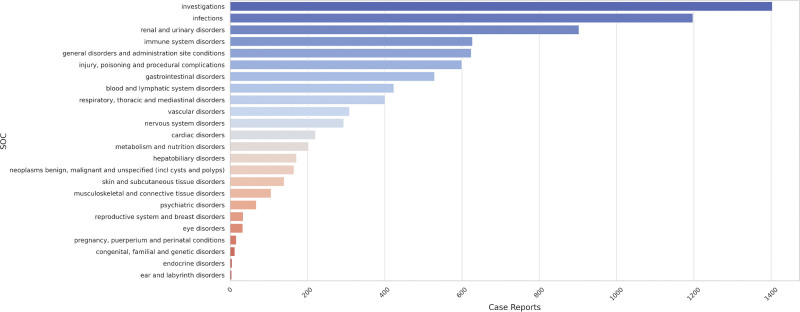
basiliximab-related case distributions across the SOCs.

The research analyzed AE reports related to basiliximab and identified AEs associated with 24 SOCs, as shown in Figure 4. The study showed that the 3 most frequent systems were investigations (n = 1403, ROR 2.84, PRR 2.54, IC 1.34, EBGM 2.54), infections and infestations (n = 1198, ROR 2.85, PRR 2.59, IC 1.37, EBGM 2.59), and renal and urinary disorders (n = 903, ROR 6.01, PRR 5.48, IC 2.45, EBGM 5.47), which is consistent with the characteristics of basiliximab as an antirejection drug. The details are presented in Table 4.

**Table 4:** The signal strength of ADEs of basiliximab at the SOC level in FAERS database.

SOC	Case reports	ROR (95% CI)	PRR (95% CI)	chisq	IC (IC025)	EBGM (EBGM05)
Investigations	1403	2.84 (2.69, 3.01)	2.54 (2.44, 2.64)	1400.02	1.34 (1.26)	2.54 (2.42)
Infections and infestations	1198	2.85 (2.68, 3.03)	2.59 (2.44, 2.75)	1234.4	1.37 (1.28)	2.59 (2.46)
Renal and urinary disorders	903	6.01 (5.61, 6.44)	5.48 (5.17, 5.81)	3367.24	2.45 (2.35)	5.47 (5.17)
Immune system disorders	627	6.85 (6.32, 7.43)	6.42 (5.94, 6.94)	2899.83	2.68 (2.56)	6.42 (5.99)
General disorders and administration site conditions	624	0.36 (0.33, 0.39)	0.4 (0.37, 0.43)	667.24	-1.3 (−1.42)	0.4 (0.38)
Injury, poisoning and procedural complications	600	0.73 (0.67, 0.8)	0.75 (0.69, 0.81)	54.06	−0.41 (−0.53)	0.75 (0.7)
Gastrointestinal disorders	529	0.68 (0.62, 0.74)	0.7 (0.65, 0.76)	76.58	−0.52 (−0.65)	0.7 (0.65)
Blood and lymphatic system disorders	424	2.92 (2.64, 3.22)	2.82 (2.56, 3.11)	506.99	1.5 (1.35)	2.82 (2.6)
Respiratory, thoracic and mediastinal disorders	401	0.94 (0.85, 1.04)	0.95 (0.86, 1.05)	1.24	−0.08 (−0.22)	0.95 (0.87)
Vascular disorders	309	1.62 (1.44, 1.81)	1.6 (1.42, 1.8)	70.2	0.67 (0.51)	1.6 (1.45)
Nervous system disorders	294	0.37 (0.33, 0.41)	0.39 (0.35, 0.44)	312.51	−1.37 (−1.53)	0.39 (0.35)
Cardiac disorders	221	0.92 (0.8, 1.05)	0.92 (0.8, 1.06)	1.61	−0.12 (−0.31)	0.92 (0.82)
Metabolism and nutrition disorders	203	1.06 (0.93, 1.22)	1.06 (0.92, 1.22)	0.76	0.09 (−0.11)	1.06 (0.95)
Hepatobiliary disorders	172	2.15 (1.85, 2.5)	2.13 (1.82, 2.49)	103.56	1.09 (0.87)	2.13 (1.87)
Neoplasms benign, malignant and unspecified (incl cysts and polyps)	165	0.68 (0.58, 0.79)	0.69 (0.59, 0.81)	24.28	−0.54 (−0.76)	0.69 (0.6)
Skin and subcutaneous tissue disorders	140	0.28 (0.24, 0.34)	0.3 (0.26, 0.35)	249.33	−1.76 (−2)	0.3 (0.26)
Musculoskeletal and connective tissue disorders	106	0.22 (0.18, 0.26)	0.23 (0.19, 0.28)	298.75	−2.15 (−2.42)	0.23 (0.19)
Psychiatric disorders	68	0.13 (0.1, 0.16)	0.13 (0.1, 0.16)	411.27	−2.92 (−3.26)	0.13 (0.11)
Reproductive system and breast disorders	34	0.46 (0.33, 0.64)	0.46 (0.33, 0.64)	22.06	−1.13 (−1.61)	0.46 (0.35)
Eye disorders	33	0.18 (0.13, 0.26)	0.19 (0.14, 0.27)	119.45	−2.42 (−2.91)	0.19 (0.14)
Pregnancy, puerperium and perinatal conditions	16	0.41 (0.25, 0.67)	0.41 (0.25, 0.67)	13.49	−1.28 (−1.97)	0.41 (0.27)
Congenital, familial and genetic disorders	12	0.43 (0.25, 0.76)	0.43 (0.24, 0.76)	8.94	−1.21 (−1.99)	0.43 (0.27)
Endocrine disorders	5	0.22 (0.09, 0.54)	0.22 (0.09, 0.53)	13.42	−2.15 (−3.31)	0.22 (0.11)
Ear and labyrinth disorders	4	0.1 (0.04, 0.28)	0.1 (0.04, 0.27)	30.91	−3.26 (−4.53)	0.1 (0.05)

ADEs = adverse drug events, chisq = chi-squared, CI = confidence interval, EBGM = empirical Bayesian geometric mean, FAERS = Food and Drug Administration Adverse Event Reporting System, IC = information component, PRR = proportional reporting ratio, ROR = reporting odds ratio, SOC = system organ classification.

#### 3.2.2. Signals detection based on PT levels

**Figure 5.**
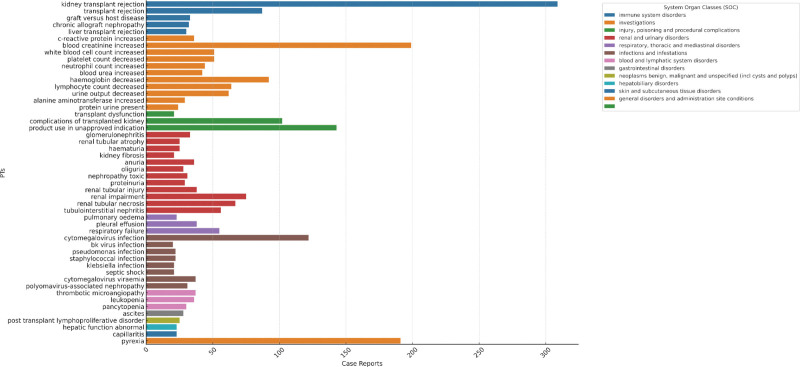
Bar graphs show the number of top 50 AEs for basiliximab in the FAERS database. The color indicates the SOC corresponding to the PT. Abscissa indicates the number of reports.

This study applied 4 algorithms at the PT level to assess drug reactions and their alignment with screening criteria, identifying 295 PTs listed in Supplementary Table S1, Supplemental Digital Content, http://links.lww.com/MD/N491. The numbers of PT reports greater than 5 are listed in Table 5. In addition, this study ranked the top 50 PTs based on the number of reports they reported, as shown in Figure 5. In this study, adverse reactions included increased blood creatinine, pyrexia, cytomegalovirus infection, decreased hemoglobin, decreased lymphocyte count, decreased urine output, respiratory failure, increased white blood cell count, decreased platelet count, and increased neutrophil count. These monitored adverse reactions were consistent with basiliximab drug instructions. Notably, this study revealed some adverse reactions beyond basiliximab drug instructions, such as mitral valve calcification, diastolic dysfunction, pelvic fluid collection, testicular swelling, soft tissue necrosis, and muscle necrosis.

**Table 5:** PTs reporting numbers >5 in the FAERS database.

SOC	PTs	Case (n)	ROR (95% CI)
Infections	Polyomavirus-associated nephropathy	31	108.15 (75.76, 154.38)
	Human herpesvirus 8 infection	6	85.77 (38.29, 192.1)
	Cytomegalovirus viraemia	37	67.55 (48.81, 93.47)
	Cytomegalovirus infection	122	51.85 (43.33, 62.05)
	BK virus infection	20	48.71 (31.35, 75.69)
	Urinary tract infection enterococcal	7	47 (22.33, 98.91)
	Klebsiella infection	21	30.15 (19.63, 46.32)
	Enterobacter infection	7	29.05 (13.82, 61.06)
	Adenovirus infection	11	28.17 (15.57, 50.96)
	Cytomegalovirus colitis	8	27.9 (13.92, 55.9)
	Fungaemia	6	27.43 (12.3, 61.19)
	Nocardiosis	9	25.6 (13.29, 49.29)
	Enterococcal infection	16	23.37 (14.3, 38.21)
	Epstein–barr virus infection	20	22.79 (14.68, 35.38)
	Mucormycosis	7	19.37 (9.22, 40.69)
	Pseudomonas infection	22	18.76 (12.34, 28.53)
	Pneumocystis jirovecii pneumonia	20	15.09 (9.72, 23.41)
	Escherichia urinary tract infection	7	10.92 (5.2, 22.92)
	Escherichia infection	8	6.96 (3.48, 13.93)
	Hepatitis c	13	6.49 (3.76, 11.18)
	Herpes simplex	6	6.46 (2.9, 14.39)
	Bronchopulmonary aspergillosis	7	6.41 (3.05, 13.46)
	Pyelonephritis	7	5.58 (2.66, 11.72)
	Wound infection	7	5.22 (2.49, 10.95)
	Urosepsis	7	5.16 (2.46, 10.83)
	Staphylococcal infection	22	4.35 (2.86, 6.61)
	Bacteremia	7	4.25 (2.02, 8.91)
	Bacterial infection	11	4.19 (2.32, 7.56)
	Candida infection	9	3.95 (2.05, 7.59)
	Septic shock	21	3.41 (2.22, 5.24)
Renal and urinary disorders	Renal tubular injury	38	332.1 (239.31, 460.87)
	Edematous kidney	12	322.45 (180.21, 576.98)
	Renal cortical necrosis	7	240.89 (113.06, 513.29)
	Renal tubular atrophy	25	197.5 (132.49, 294.41)
	Renal arteriosclerosis	6	175.91 (78.05, 396.46)
	Renal vein thrombosis	12	135.57 (76.45, 240.4)
	Ureteric stenosis	15	128.98 (77.28, 215.25)
	Glomerulosclerosis	12	125.01 (70.53, 221.56)
	Renal hematoma	9	115.74 (59.81, 223.98)
	Glomerulonephritis	33	98.41 (69.71, 138.92)
	Kidney fibrosis	21	82.57 (53.64, 127.1)
	IgA nephropathy	14	72.81 (42.96, 123.39)
	Focal segmental glomerulosclerosis	16	72.42 (44.21, 118.63)
	Renal tubular necrosis	67	47.12 (37.02, 59.99)
	Nephrosclerosis	8	43.01 (21.45, 86.26)
	Renal hemorrhage	10	32.03 (17.2, 59.65)
	Oliguria	28	29.81 (20.55, 43.24)
	Renal tubular disorder	12	27.25 (15.45, 48.07)
	Anuria	36	26.88 (19.36, 37.32)
	Tubulointerstitial nephritis	56	20.85 (16.03, 27.13)
	Nephropathy toxic	31	20.41 (14.34, 29.06)
	Hydronephrosis	19	17.16 (10.93, 26.94)
	Azotemia	8	13.93 (6.96, 27.88)
	Cystitis hemorrhagic	7	12.18 (5.8, 25.57)
	Proteinuria	29	11.05 (7.67, 15.92)
	Polyuria	11	9 (4.98, 16.26)
	Renal impairment	75	6.26 (4.99, 7.86)
	Hematuria	25	4.75 (3.2, 7.03)
	Nephropathy	6	4.12 (1.85, 9.18)
Investigations	Red blood cells urine positive	19	127 (80.57, 200.19)
	Urine output decreased	62	46.58 (36.25, 59.86)
	Cytomegalovirus test positive	10	38.87 (20.86, 72.42)
	Immunosuppressant drug level decreased	6	33.8 (15.15, 75.44)
	Protein urine present	24	32.97 (22.06, 49.27)
	Neutrophil count increased	44	28.53 (21.2, 38.4)
	Lymphocyte count decreased	64	24.37 (19.05, 31.18)
	Immunosuppressant drug level increased	10	22.23 (11.94, 41.38)
	Blood creatinine increased	199	20.49 (17.8, 23.59)
	Blood creatinine decreased	8	17.65 (8.81, 35.34)
	Bilirubin conjugated increased	6	17.09 (7.67, 38.1)
	Blood albumin decreased	20	16.71 (10.77, 25.94)
	Blood urea increased	42	15.79 (11.65, 21.39)
	Urine output increased	6	14.92 (6.69, 33.25)
	High density lipoprotein decreased	7	13.5 (6.43, 28.36)
	White blood cell count increased	51	8.69 (6.6, 11.45)
	Low density lipoprotein increased	8	7.41 (3.7, 14.84)
	Blood uric acid increased	6	7.2 (3.23, 16.05)
	C-reactive protein increased	36	6.96 (5.02, 9.66)
	Blood lactate dehydrogenase increased	16	6.3 (3.85, 10.28)
	Prothrombin time prolonged	6	6.02 (2.7, 13.41)
	Hemoglobin decreased	92	5.95 (4.85, 7.31)
	Hematocrit decreased	14	4.44 (2.63, 7.5)
	Blood urine present	10	3.45 (1.86, 6.42)
	Transaminases increased	11	3.32 (1.83, 5.99)
	Platelet count decreased	51	3.25 (2.46, 4.27)
	Alanine aminotransferase increased	29	3.1 (2.16, 4.47)
Injury, poisoning and procedural complications	Complications of transplanted kidney	102	321.66 (263.22, 393.08)
	Delayed graft function	19	196.67 (124.43, 310.84)
	Graft loss	12	108.5 (61.27, 192.14)
	Incision site pain	18	102.35 (64.19, 163.2)
	Transplant dysfunction	21	83.54 (54.27, 128.59)
	Graft complication	7	81.27 (38.53, 171.42)
	Renal transplant failure	7	52.72 (25.04, 111.01)
	Transplant failure	12	33.96 (19.25, 59.92)
	Wound complication	7	13.74 (6.54, 28.85)
	Wound dehiscence	7	13.54 (6.45, 28.43)
	Product use in unapproved indication	143	4.75 (4.03, 5.61)
	Post procedural complication	11	3.76 (2.08, 6.79)
	Procedural pain	14	3.62 (2.14, 6.11)
Immune system disorders	Intestine transplant rejection	12	1169.58 (628.51, 2176.44)
	Kidney transplant rejection	309	468.99 (416.86, 527.63)
	Pancreas transplant rejection	12	445.19 (247.36, 801.27)
	Chronic allograft nephropathy	32	289.56 (202.88, 413.26)
	Liver transplant rejection	30	116.41 (81.05, 167.21)
	Transplant rejection	87	76.54 (61.88, 94.67)
	Lung transplant rejection	7	73.81 (35.01, 155.62)
	Heart transplant rejection	6	39.4 (17.65, 87.97)
	Graft versus host disease in skin	10	32.32 (17.35, 60.19)
	Graft versus host disease in gastrointestinal tract	9	30.54 (15.86, 58.82)
	Graft versus host disease	33	30.54 (21.68, 43.02)
	Anaphylactic shock	16	4.45 (2.73, 7.28)
Vascular disorders	Lymphocele	12	112.02 (63.24, 198.42)
	Arteritis	8	85.15 (42.35, 171.2)
	Venous thrombosis	6	10.01 (4.49, 22.31)
	Ischemia	7	9.62 (4.58, 20.19)
	Haemodynamic instability	8	7.66 (3.83, 15.32)
	Shock	16	4.89 (2.99, 7.99)
Blood and lymphatic system disorders	Lymphocytic infiltration	17	113.47 (70.18, 183.47)
	Thrombotic microangiopathy	37	28.54 (20.65, 39.45)
	Hemolytic uremic syndrome	9	23.97 (12.45, 46.15)
	Hemolytic anemia	11	8.16 (4.51, 14.74)
	Disseminated intravascular coagulation	12	5.49 (3.11, 9.67)
	Leukopenia	36	4.95 (3.57, 6.86)
	Splenomegaly	8	4.54 (2.27, 9.09)
	Coagulopathy	11	4.32 (2.39, 7.81)
	Lymphopenia	8	3.92 (1.96, 7.85)
	Pancytopenia	30	3.73 (2.61, 5.35)
Hepatobiliary disorders	Hepatic necrosis	10	16.72 (8.98, 31.11)
	Cholangitis	7	8.38 (3.99, 17.6)
	Hepatitis cholestatic	6	6.98 (3.13, 15.56)
	Hepatic function abnormal	23	4.38 (2.91, 6.6)
	Cholestasis	10	3.64 (1.96, 6.78)
	Hepatic failure	15	3.28 (1.98, 5.45)
Respiratory, thoracic and mediastinal disorders	Noncardiogenic pulmonary edema	6	48.15 (21.55, 107.57)
	Acute pulmonary edema	8	9.35 (4.67, 18.71)
	Acute respiratory distress syndrome	19	7.25 (4.62, 11.37)
	Lung infiltration	10	6.92 (3.72, 12.88)
	Respiratory failure	55	5.04 (3.87, 6.57)
	Pleural effusion	38	4.15 (3.02, 5.71)
	Atelectasis	6	3.97 (1.78, 8.84)
	Pneumothorax	9	3.89 (2.02, 7.47)
	Pulmonary edema	23	3.41 (2.27, 5.14)
	Respiratory distress	14	3.37 (2, 5.7)
Gastrointestinal disorders	Ascites	28	6.39 (4.41, 9.26)
	Large intestine perforation	6	5.82 (2.61, 12.96)
General disorders and administration site conditions	Systemic inflammatory response syndrome	7	12 (5.71, 25.19)
	Multiple organ dysfunction syndrome	17	4.78 (2.97, 7.69)
	Pyrexia	191	3.77 (3.27, 4.35)
Neoplasms benign, malignant and unspecified (incl cysts and polyps)	Kaposi sarcoma	19	42.36 (26.96, 66.56)
	Post transplant lymphoproliferative disorder	25	38.52 (25.98, 57.11)
	Diffuse large b-cell lymphoma	17	17.35 (10.77, 27.94)
	Lymphoproliferative disorder	6	14.4 (6.46, 32.08)
Nervous system disorders	Leukoencephalopathy	6	10.56 (4.74, 23.52)
	Posterior reversible encephalopathy syndrome	13	9.96 (5.78, 17.17)
	Hemiplegia	8	6.25 (3.12, 12.5)
	Brain edema	7	3.73 (1.78, 7.83)
Metabolism and nutrition disorders	Hyperuricemia	9	14.9 (7.75, 28.68)
	Hypoalbuminaemia	8	7.32 (3.66, 14.64)
	Hyperkalaemia	18	3.51 (2.21, 5.58)
Cardiac disorders	Left ventricular hypertrophy	7	10.58 (5.04, 22.22)
Skin and subcutaneous tissue disorders	Capillaritis	23	980.98 (630.11, 1527.21)

CI = confidence interval, FAERS = Food and Drug Administration Adverse Event Reporting System, PTs = preferred terms, ROR = reporting odds ratio, SOC = system organ classification.

### 3.3. Onset time of events

**Figure 6.**
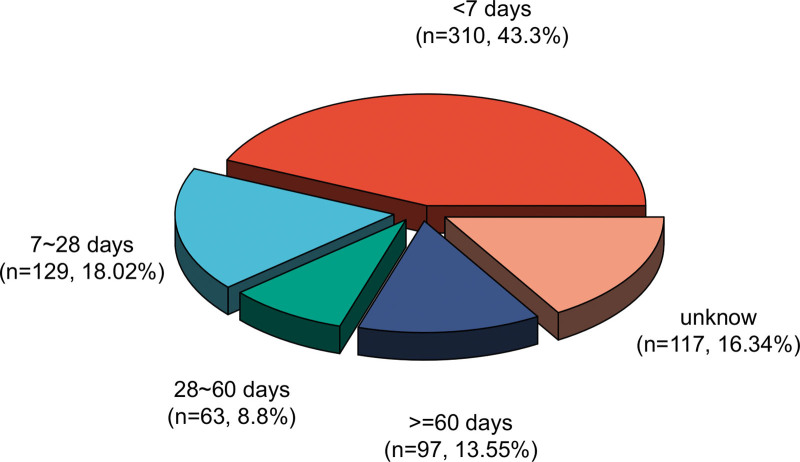
Time to onset of basiliximab-related adverse AEs.

Figure 6 shows the timing of basiliximab-related AE. A total of 439 AEs (68.32 %) occurred within 28 days after drug administration, of which 310 (43.30%) occurred within 1 week of drug administration. However, a subset of AEs (n = 97, 13.55%) occurred after long-term (> 2 months) treatment with basiliximab. Notably, there was a large proportion of adverse reactions (n = 117, 16.34%), and the time of occurrence was unknown.

### 3.4. Concomitant medication

Table 6 meticulously enumerates the most frequently used concomitant medications in conjunction with basiliximab, with a focus on those reported in AE scenarios. The enumeration highlights the top ten concomitant medications, which notably include prednisolone, with a considerable frequency of 719 instances, followed by tacrolimus (454 times) and mycophenolate mofetil (388 times). Additionally, furosemide was mentioned in 362 reports, ganciclovir in 207, sulfamethoxazole in 170, sodium bicarbonate in 154, prednisone in 150, nifedipine in 138, and albumin in 120.

**Table 6:** Top 30 concomitant medications.

Rank	Concomitant medication	Count
1	Prednisolone	719
2	Tacrolimus	454
3	Mycophenolate mofetil	388
4	Furosemide	362
5	Ganciclovir	207
6	Sulfamethoxazole	170
7	Sodium bicarbonate	154
8	Prednisone	150
9	Nifedipine	138
10	Albumin	120
11	Insulin	101
12	Calcium gluconate	94
13	Cyclosporine	93
14	Erythropoietin	85
15	Ambroxol hydrochloride	82
16	Acetaminophen	76
17	Alprostadil	75
18	Torsemide	72
19	Aspirin	71
20	Esomeprazole magnesium	59
21	Metoprolol tartrate	59
22	Ranitidine hydrochloride	53
23	Hydrotalcite	51
24	Lansoprazole	50
25	Rituximab	48
26	Diltiazem hydrochloride	47
27	Folic acid	40
28	Acyclovir	39
29	Meropenem	38
30	Amikacin	38

Further analysis extends to the top 30 drugs, which are systematically categorized into several groups based on their therapeutic applications. These categories include immunosuppressants, such as tacrolimus, mycophenolate mofetil, prednisolone, prednisone, cyclosporine, and rituximab, which play a pivotal role in preventing organ rejection. The diuretic category includes furosemide and torsemide, which are essential for managing fluid balance. The antiviral agents listed are ganciclovir and acyclovir, which are crucial for combating viral infections in immunocompromised patients. Antibiotics include potent drugs, such as sulfamethoxazole, meropenem, and amikacin, which are integral to the prevention and treatment of bacterial infections.

Moreover, the list includes categories such as electrolytes and minerals, represented by sodium bicarbonate, calcium gluconate, and albumin, which are vital for maintaining the electrolyte balance and plasma volume. Hormones and related therapeutic agents such as insulin, erythropoietin, and folic acid are crucial for metabolic regulation and anemia, respectively. Respiratory drugs, including ambroxol hydrochloride, are beneficial for managing respiratory conditions. The analgesics and antipyretics category, featuring acetaminophen and aspirin, addresses pain and fever management.

Cardiovascular medications, such as nifedipine, metoprolol tartrate, and diltiazem hydrochloride, are essential for managing blood pressure and heart rhythm disorders. The gastrointestinal drugs listed include esomeprazole magnesium, ranitidine hydrochloride, lansoprazole, and hydrotalcite, all of which help manage acid-related gastrointestinal conditions. Lastly, the vasodilators category, with drugs such as alprostadil, plays a crucial role in improving blood flow. This comprehensive categorization underscores the complex and multidimensional nature of medication management in basiliximab-treated patients.

## 4. Discussion

To our knowledge, this is the most thorough pharmacovigilance study to date on basiliximab-associated AEs using postmarketing data from the FAERS database. This study offers a precise and comprehensive description of the AEs associated with basiliximab. Real-world monitoring of medication uses and associated AEs aftermarket release is crucial for confirming safety and efficacy. By analyzing FAERS data from Q1 2004 to Q4 2023, this study not only corroborated existing safety information, but also uncovered potential new risks, providing valuable insights for clinical practice and public health policy. An in-depth analysis is as follows.

The disparity in basiliximab-associated AEs between sexes was evident, yet the substantial lack of sex data (12.17%) hindered a precise analysis of sex-specific reactions. Future efforts should prioritize the collection of sex-specific data to enhance our understanding of the adverse effects of basiliximab. The scarcity of age-related information also limits insights into AEs across different age groups, underscoring the need for more detailed age data in future research to examine age-related drug responses. The low rate of physician-reported cases (28.49%) points to a gap in physician engagement with AE reporting; thus, increasing physician awareness and reporting is crucial. With 10.92% of reports coming from India and 10.72% reporting from the USA, further research is needed to explore the potential regional or cultural factors influencing these findings.

The most common adverse reactions monitored in this study included increased serum creatinine level, fever, cytomegalovirus infection, decreased hemoglobin level, decreased lymphocyte count, decreased urine output, respiratory failure, increased white blood cell count, decreased platelet count, and decreased neutrophil count. In addition, we also found that bacterial sepsis, fungal skin infection, abdominal abscess, subcutaneous abscess, Aspergillus infection, renal artery thrombosis, and renal atrophy only occurred in a small number of patients. The most common SOCs are investigations, infections, renal and urinary disorders, immune system disorders, general disorders, administration site conditions, injuries, poisoning and procedural complications, gastrointestinal disorders, blood and lymphatic system disorders, and respiratory, thoracic, and mediastinal disorders. These results are generally consistent with the manufacturer’s labeling and clinical trials.

Research demonstrates that there are no significant disparities in infection rates between patients administered basiliximab and those administered a placebo, as documented in multiple studies.^[3,4,16,17]^ This indicates that basiliximab, used in this context, does not increase the general risk of infections compared with standard care without active treatment. However, it is important to note that urinary tract infections continue to be frequently observed among individuals receiving basiliximab, suggesting a specific area where this medication does not reduce the infection prevalence.^[3,16]^ In a robust study involving a large cohort of 722 renal transplant recipients, which included both individuals with and without diabetes, basiliximab demonstrated a high level of tolerability across this diverse group of patients.^[18]^ Furthermore, detailed comparisons between basiliximab and antithymocyte globulin, another commonly used induction therapy agent, indicated that both treatments offer comparable safety outcomes.

Studies show that neither treatment significantly increases the risk of developing malignancies or infections or leads to higher mortality rates.^[19,20]^ Notably, basiliximab is associated with a lower incidence of AEs, with only 11% of patients experiencing undesirable effects as opposed to 42% of those treated with antithymocyte globulin.^[19]^ This significant difference highlights the advantage of basiliximab in terms of patient experience and risk management, making it a potentially more favorable option in clinical settings, where the reduction of adverse effects is a critical concern. This reinforces the role of basiliximab as a preferred agent in transplantation medicine, balancing its effectiveness with patient safety.

A focused study involving 32 kidney transplant recipients further reinforced the notion that basiliximab is well tolerated, with negligible immune reactions observed in the participants.^[21]^ Despite its overall safe profile, there have been instances where acute hypersensitivity reactions to basiliximab, as well as to daclizumab, another immunosuppressive drug, have been reported. These reactions are rare, but serious enough to warrant attention. Nevertheless, when compared overall, the side effects of both basiliximab and daclizumab are largely similar to those observed with placebo, indicating that these reactions are exceptions rather than the rule.^[4,22]^ The side effects associated with these immunosuppressive agents affect only a small percentage of recipients, ranging from 3% to 10%. These primarily include conditions related to dermatological issues and infections, which, while relatively minor, require monitoring and management to ensure patient safety and comfort.^[4]^

Recent research has shown that the typical side effects associated with basiliximab are diverse and include symptoms such as chills, fever, rash, fatigue, diarrhea, nausea, headache, anorexia, leukopenia, and infections. These side effects are relatively common and are recognized as manageable within the context of treatment, although they can significantly affect patient comfort and treatment adherence.^[23]^

In addition to these common adverse effects, this study also highlights a spectrum of rare but potentially severe side effects associated with the use of basiliximab. These include acute allergic reactions, which can manifest swiftly and intensely; anaphylaxis, a life-threatening systemic allergic reaction that requires immediate medical intervention; capillary leak syndrome, which involves a sudden leakage of fluid from the capillaries that can lead to shock; cytokine release syndrome, a severe inflammatory response that can be life-threatening; and progressive multifocal leukoencephalopathy, a rare brain infection that leads to significant neurological decline.^[23]^ Awareness and prompt management of these severe side effects are crucial to ensure patient safety and optimize treatment outcomes.

The data observed at this time, based on the FAERS database, show that among all reported infectious diseases, there were only 3 cases of urinary tract infections, and the most common infectious disease was cytomegalovirus infection (n = 122). This reminds us that, among the adverse reactions of basiliximab, attention should be paid to the adverse reactions of infection, especially cytomegalovirus infection.

It is worth noting that no acute AEs of hypersensitivity reactions to basiliximab were observed in previous studies.^[3,4,21,24,25]^ However, this study found that anaphylactic shock had a high frequency and signal intensity (n = 16, ROR 4.45, PRR 4.45, IC 2.15, EBGM 4.45). This reminds us to pay attention to monitor the occurrence of anaphylactic shock when basiliximab is used.

A total of 439 AEs (68.32 %) occurred within 28 days after drug administration, of which 310 (43.30%) occurred within 1 week of drug administration. However, 97 AEs (13.55 %) occurred after long-term (>2 months) basiliximab treatment. This may be due to the latent period of the drug, or because the patient’s body has strong resistance and slow metabolism, which prolongs the time required to establish the cumulative drug concentration, and the body cannot fully absorb the drug, so most adverse drug reactions occur later. Therefore, a longer follow-up period is required to observe the adverse effects of basiliximab in future clinical studies.

The described AEs and their potential mechanisms associated with basiliximab remain speculative. Confirming the exact causes requires further investigation, considering the influence of drug properties, individual variability, and preexisting conditions on AE development. Therefore, additional clinical and experimental studies are essential for a comprehensive understanding of AEs. Clinicians should closely monitor AE occurrence to manage them effectively.

Our study of the safety of basiliximab, while providing valuable insights, has limitations. First, reliance on voluntary FAERS reporting could lead to skewed data due to factors such as FDA warnings, possibly causing underreporting or inaccuracies. Second, our analysis offers a statistical perspective on potential risks without confirming the causal links between the drug and reported AEs. Incomplete patient information in some reports further complicates causality assessments, highlighting the need for additional research. Despite these challenges, this study contributes to the understanding of basiliximab-related AEs, supporting their cautious use in healthcare. Further investigation is necessary to fully decode AE mechanisms. Healthcare professionals should monitor AEs to effectively manage risks.

## 5. Conclusion

In conclusion, although this study illuminates the safety profile of basiliximab through extensive pharmacovigilance analysis, it is important to recognize its inherent limitations. Dependency on voluntary FAERS reports introduces potential biases, including underreporting and the influence of external alerts, which may skew the findings. Furthermore, the study’s analytical approach, focused on signal strength, stops short of establishing causality between basiliximab and the observed AEs. Despite these challenges, our research provides valuable insights into the spectrum of AEs associated with basiliximab, enhancing our collective understanding and guiding safer clinical use. These findings underscore the necessity for ongoing vigilance in monitoring AEs and advocate for further research to elucidate the underlying mechanisms of these events. Through these efforts, we can better navigate the complexities of drug safety and optimize patient care in the evolving field of organ transplantation.

## Author contributions

**Conceptualization:** Sheng Chen, Xiaohan Ma, Jianqiang Zhang.

**Data curation:** Sheng Chen, Xiaohan Ma, Jianqiang Zhang.

**Formal analysis:** Sheng Chen, Xiaohan Ma, Jianqiang Zhang.

**Funding acquisition:** Xiaohan Ma, Jianqiang Zhang.

**Investigation:** Sheng Chen, Xiaohan Ma, Jianqiang Zhang.

**Methodology:** Xiaohan Ma, Jianqiang Zhang.

**Project administration:** Sheng Chen, Xiaohan Ma, Jianqiang Zhang.

**Resources:** Jianqiang Zhang.

**Software:** Sheng Chen, Xiaohan Ma, Jianqiang Zhang.

**Supervision:** Sheng Chen, Xiaohan Ma, Jianqiang Zhang.

**Validation:** Sheng Chen.

**Visualization:** Sheng Chen, Xiaohan Ma.

**Writing – original draft:** Sheng Chen, Xiaohan Ma.

**Writing – review & editing:** Sheng Chen, Xiaohan Ma, Jianqiang Zhang.

## Supplementary Material


