# Supplementary material for: Safety assessment of basiliximab using real-world adverse event data from the FDA Adverse Event Reporting System Database: A retrospective observational study
Source: Medicine (Baltimore). 2024 Sep 6;103(36):e39537. doi: 10.1097/MD.0000000000039537 (PMC11384058; doi:10.1097/MD.0000000000039537)
Supplement: Supplementary file 1 [file medi-103-e39537-s001.docx]

**Supplementary Material**

**Table S 1.** All reports of basiliximab-related adverse reactions at the PT level.

| SOC | PT | Case Reports | ROR(95% CI) | PRR(95% CI) | chisq | IC(IC025) | EBGM(EBGM05) |
| --- | --- | --- | --- | --- | --- | --- | --- |
| immune system disorders | kidney transplant rejection | 309 | 468.99(416.86, 527.63) | 451.96(401.82, 508.36) | 128903.5 | 8.71(8.54) | 419.05(379.71) |
| investigations | blood creatinine increased | 199 | 20.49(17.8, 23.59) | 20.03(17.46, 22.98) | 3590.48 | 4.32(4.12) | 19.97(17.75) |
| general disorders and administration site conditions | pyrexia | 191 | 3.77(3.27, 4.35) | 3.71(3.23, 4.26) | 379.81 | 1.89(1.68) | 3.71(3.29) |
| injury, poisoning and procedural complications | product use in unapproved indication | 143 | 4.75(4.03, 5.61) | 4.69(4.01, 5.49) | 416.31 | 2.23(1.99) | 4.69(4.08) |
| infections and infestations | cytomegalovirus infection | 122 | 51.85(43.33, 62.05) | 51.12(42.85, 60.98) | 5943.94 | 5.66(5.41) | 50.68(43.61) |
| injury, poisoning and procedural complications | complications of transplanted kidney | 102 | 321.66(263.22, 393.08) | 317.81(261.24, 386.62) | 30524.48 | 8.23(7.95) | 301.19(254.67) |
| investigations | haemoglobin decreased | 92 | 5.95(4.85, 7.31) | 5.9(4.85, 7.18) | 374.74 | 2.56(2.26) | 5.9(4.96) |
| immune system disorders | transplant rejection | 87 | 76.54(61.88, 94.67) | 75.76(61.07, 93.99) | 6335.83 | 6.22(5.92) | 74.79(62.6) |
| renal and urinary disorders | renal impairment | 75 | 6.26(4.99, 7.86) | 6.22(4.92, 7.87) | 328.52 | 2.64(2.31) | 6.21(5.14) |
| renal and urinary disorders | renal tubular necrosis | 67 | 47.12(37.02, 59.99) | 46.76(36.96, 59.16) | 2976.58 | 5.54(5.19) | 46.39(37.91) |
| investigations | lymphocyte count decreased | 64 | 24.37(19.05, 31.18) | 24.19(19.12, 30.6) | 1417.44 | 4.59(4.24) | 24.1(19.61) |
| investigations | urine output decreased | 62 | 46.58(36.25, 59.86) | 46.25(35.85, 59.67) | 2723.28 | 5.52(5.16) | 45.89(37.2) |
| renal and urinary disorders | tubulointerstitial nephritis | 56 | 20.85(16.03, 27.13) | 20.72(16.06, 26.73) | 1047.64 | 4.37(3.99) | 20.65(16.57) |
| respiratory, thoracic and mediastinal disorders | respiratory failure | 55 | 5.04(3.87, 6.57) | 5.01(3.88, 6.46) | 176.84 | 2.33(1.95) | 5.01(4.01) |
| investigations | white blood cell count increased | 51 | 8.69(6.6, 11.45) | 8.64(6.57, 11.37) | 344.47 | 3.11(2.72) | 8.63(6.86) |
| investigations | platelet count decreased | 51 | 3.25(2.46, 4.27) | 3.23(2.45, 4.25) | 78.7 | 1.69(1.3) | 3.23(2.57) |
| investigations | neutrophil count increased | 44 | 28.53(21.2, 38.4) | 28.39(21.16, 38.09) | 1157.3 | 4.82(4.4) | 28.26(22.04) |
| investigations | blood urea increased | 42 | 15.79(11.65, 21.39) | 15.71(11.71, 21.08) | 577.3 | 3.97(3.54) | 15.67(12.16) |
| renal and urinary disorders | renal tubular injury | 38 | 332.1(239.31, 460.87) | 330.62(236.93, 461.36) | 11807.95 | 8.29(7.82) | 312.67(237.69) |
| respiratory, thoracic and mediastinal disorders | pleural effusion | 38 | 4.15(3.02, 5.71) | 4.14(3.03, 5.66) | 90.49 | 2.05(1.59) | 4.14(3.17) |
| infections and infestations | cytomegalovirus viraemia | 37 | 67.55(48.81, 93.47) | 67.26(48.2, 93.86) | 2387.27 | 6.06(5.59) | 66.49(50.67) |
| blood and lymphatic system disorders | thrombotic microangiopathy | 37 | 28.54(20.65, 39.45) | 28.42(20.77, 38.89) | 974.16 | 4.82(4.36) | 28.28(21.57) |
| renal and urinary disorders | anuria | 36 | 26.88(19.36, 37.32) | 26.77(19.18, 37.36) | 889.21 | 4.74(4.27) | 26.65(20.26) |
| investigations | c-reactive protein increased | 36 | 6.96(5.02, 9.66) | 6.93(4.97, 9.67) | 182.69 | 2.79(2.33) | 6.93(5.27) |
| blood and lymphatic system disorders | leukopenia | 36 | 4.95(3.57, 6.86) | 4.93(3.53, 6.88) | 112.78 | 2.3(1.83) | 4.93(3.75) |
| renal and urinary disorders | glomerulonephritis | 33 | 98.41(69.71, 138.92) | 98.03(68.89, 139.5) | 3116.33 | 6.59(6.1) | 96.4(72.25) |
| immune system disorders | graft versus host disease | 33 | 30.54(21.68, 43.02) | 30.42(21.8, 42.45) | 934.17 | 4.92(4.43) | 30.27(22.72) |
| immune system disorders | chronic allograft nephropathy | 32 | 289.56(202.88, 413.26) | 288.47(202.71, 410.51) | 8728.73 | 8.1(7.6) | 274.72(204) |
| infections and infestations | polyomavirus-associated nephropathy | 31 | 108.15(75.76, 154.38) | 107.76(75.72, 153.35) | 3218.43 | 6.73(6.22) | 105.79(78.54) |
| renal and urinary disorders | nephropathy toxic | 31 | 20.41(14.34, 29.06) | 20.34(14.29, 28.94) | 568.21 | 4.34(3.84) | 20.27(15.09) |
| immune system disorders | liver transplant rejection | 30 | 116.41(81.05, 167.21) | 116.01(81.52, 165.09) | 3352.83 | 6.83(6.32) | 113.73(84) |
| blood and lymphatic system disorders | pancytopenia | 30 | 3.73(2.61, 5.35) | 3.73(2.62, 5.31) | 59.83 | 1.9(1.39) | 3.72(2.76) |
| renal and urinary disorders | proteinuria | 29 | 11.05(7.67, 15.92) | 11.02(7.59, 15.99) | 263.8 | 3.46(2.94) | 11(8.11) |
| investigations | alanine aminotransferase increased | 29 | 3.1(2.16, 4.47) | 3.1(2.14, 4.5) | 41.18 | 1.63(1.11) | 3.1(2.28) |
| renal and urinary disorders | oliguria | 28 | 29.81(20.55, 43.24) | 29.71(20.47, 43.12) | 773.03 | 4.89(4.36) | 29.57(21.66) |
| gastrointestinal disorders | ascites | 28 | 6.39(4.41, 9.26) | 6.37(4.39, 9.24) | 126.63 | 2.67(2.14) | 6.36(4.66) |
| renal and urinary disorders | renal tubular atrophy | 25 | 197.5(132.49, 294.41) | 196.92(133.06, 291.43) | 4711.74 | 7.57(7.01) | 190.43(136.35) |
| renal and urinary disorders | haematuria | 25 | 4.75(3.2, 7.03) | 4.74(3.2, 7.01) | 73.64 | 2.24(1.69) | 4.73(3.41) |
| neoplasms benign, malignant and unspecified (incl cysts and polyps) | post-transplant lymphoproliferative disorder | 25 | 38.52(25.98, 57.11) | 38.41(25.95, 56.84) | 904.8 | 5.25(4.7) | 38.16(27.44) |
| investigations | protein urine present | 24 | 32.97(22.06, 49.27) | 32.88(22.22, 48.66) | 737.59 | 5.03(4.46) | 32.69(23.36) |
| hepatobiliary disorders | hepatic function abnormal | 23 | 4.38(2.91, 6.6) | 4.37(2.9, 6.6) | 59.82 | 2.13(1.55) | 4.37(3.1) |
| respiratory, thoracic and mediastinal disorders | pulmonary oedema | 23 | 3.41(2.27, 5.14) | 3.41(2.26, 5.15) | 39.11 | 1.77(1.19) | 3.41(2.42) |
| skin and subcutaneous tissue disorders | capillaritis | 23 | 980.98(630.11, 1527.21) | 978.32(623.31, 1535.54) | 19186.65 | 9.71(9.09) | 836.05(577.27) |
| infections and infestations | pseudomonas infection | 22 | 18.76(12.34, 28.53) | 18.72(12.4, 28.25) | 367.83 | 4.22(3.63) | 18.66(13.14) |
| infections and infestations | staphylococcal infection | 22 | 4.35(2.86, 6.61) | 4.34(2.88, 6.55) | 56.52 | 2.12(1.53) | 4.34(3.06) |
| infections and infestations | klebsiella infection | 21 | 30.15(19.63, 46.32) | 30.08(19.54, 46.3) | 587.34 | 4.9(4.3) | 29.93(20.9) |
| infections and infestations | septic shock | 21 | 3.41(2.22, 5.24) | 3.41(2.22, 5.25) | 35.73 | 1.77(1.16) | 3.41(2.38) |
| renal and urinary disorders | kidney fibrosis | 21 | 82.57(53.64, 127.1) | 82.37(53.52, 126.78) | 1664.19 | 6.34(5.74) | 81.22(56.61) |
| injury, poisoning and procedural complications | transplant dysfunction | 21 | 83.54(54.27, 128.59) | 83.34(54.15, 128.27) | 1683.94 | 6.36(5.75) | 82.16(57.27) |
| infections and infestations | bk virus infection | 20 | 48.71(31.35, 75.69) | 48.6(31.58, 74.8) | 924.67 | 5.59(4.97) | 48.2(33.34) |
| infections and infestations | epstein-barr virus infection | 20 | 22.79(14.68, 35.38) | 22.74(14.77, 35) | 414.12 | 4.5(3.88) | 22.66(15.68) |
| infections and infestations | pneumocystis jirovecii pneumonia | 20 | 15.09(9.72, 23.41) | 15.05(9.78, 23.16) | 261.72 | 3.91(3.29) | 15.02(10.4) |
| investigations | blood albumin decreased | 20 | 16.71(10.77, 25.94) | 16.68(10.84, 25.67) | 293.9 | 4.06(3.44) | 16.63(11.51) |
| renal and urinary disorders | hydronephrosis | 19 | 17.16(10.93, 26.94) | 17.13(10.91, 26.89) | 287.67 | 4.09(3.46) | 17.08(11.71) |
| investigations | red blood cells urine positive | 19 | 127(80.57, 200.19) | 126.72(80.74, 198.9) | 2318.67 | 6.95(6.31) | 124(84.73) |
| injury, poisoning and procedural complications | delayed graft function | 19 | 196.67(124.43, 310.84) | 196.23(125.02, 308) | 3568.57 | 7.57(6.92) | 189.78(129.39) |
| respiratory, thoracic and mediastinal disorders | acute respiratory distress syndrome | 19 | 7.25(4.62, 11.37) | 7.23(4.61, 11.35) | 101.98 | 2.85(2.22) | 7.23(4.96) |
| neoplasms benign, malignant and unspecified (incl cysts and polyps) | kaposi's sarcoma | 19 | 42.36(26.96, 66.56) | 42.27(26.93, 66.35) | 760.06 | 5.39(4.76) | 41.97(28.76) |
| injury, poisoning and procedural complications | incision site pain | 18 | 102.35(64.19, 163.2) | 102.14(63.81, 163.49) | 1771.16 | 6.65(5.99) | 100.37(67.93) |
| metabolism and nutrition disorders | hyperkalaemia | 18 | 3.51(2.21, 5.58) | 3.51(2.19, 5.62) | 32.25 | 1.81(1.16) | 3.51(2.38) |
| blood and lymphatic system disorders | lymphocytic infiltration | 17 | 113.47(70.18, 183.47) | 113.25(70.75, 181.27) | 1854.81 | 6.8(6.12) | 111.08(74.3) |
| general disorders and administration site conditions | multiple organ dysfunction syndrome | 17 | 4.78(2.97, 7.69) | 4.77(2.98, 7.64) | 50.61 | 2.25(1.59) | 4.77(3.2) |
| neoplasms benign, malignant and unspecified (incl cysts and polyps) | diffuse large b-cell lymphoma | 17 | 17.35(10.77, 27.94) | 17.32(10.82, 27.72) | 260.63 | 4.11(3.44) | 17.27(11.59) |
| infections and infestations | enterococcal infection | 16 | 23.37(14.3, 38.21) | 23.33(14.29, 38.08) | 340.62 | 4.54(3.85) | 23.24(15.4) |
| renal and urinary disorders | focal segmental glomerulosclerosis | 16 | 72.42(44.21, 118.63) | 72.29(44.29, 118) | 1110.86 | 6.16(5.47) | 71.4(47.25) |
| investigations | blood lactate dehydrogenase increased | 16 | 6.3(3.85, 10.28) | 6.29(3.85, 10.27) | 71.06 | 2.65(1.96) | 6.28(4.17) |
| immune system disorders | anaphylactic shock | 16 | 4.45(2.73, 7.28) | 4.45(2.73, 7.26) | 42.75 | 2.15(1.47) | 4.45(2.95) |
| vascular disorders | shock | 16 | 4.89(2.99, 7.99) | 4.88(2.99, 7.97) | 49.37 | 2.29(1.6) | 4.88(3.24) |
| renal and urinary disorders | ureteric stenosis | 15 | 128.98(77.28, 215.25) | 128.75(77.34, 214.32) | 1859.72 | 6.98(6.26) | 125.95(82.05) |
| hepatobiliary disorders | hepatic failure | 15 | 3.28(1.98, 5.45) | 3.28(1.97, 5.46) | 23.74 | 1.71(1) | 3.28(2.14) |
| renal and urinary disorders | iga nephropathy | 14 | 72.81(42.96, 123.39) | 72.69(42.82, 123.4) | 977.47 | 6.17(5.43) | 71.79(46.17) |
| investigations | haematocrit decreased | 14 | 4.44(2.63, 7.5) | 4.43(2.61, 7.52) | 37.18 | 2.15(1.42) | 4.43(2.86) |
| injury, poisoning and procedural complications | procedural pain | 14 | 3.62(2.14, 6.11) | 3.61(2.13, 6.13) | 26.44 | 1.85(1.12) | 3.61(2.33) |
| respiratory, thoracic and mediastinal disorders | respiratory distress | 14 | 3.37(2, 5.7) | 3.37(1.99, 5.72) | 23.35 | 1.75(1.02) | 3.37(2.17) |
| infections and infestations | hepatitis c | 13 | 6.49(3.76, 11.18) | 6.48(3.74, 11.22) | 60.15 | 2.69(1.94) | 6.47(4.1) |
| nervous system disorders | posterior reversible encephalopathy syndrome | 13 | 9.96(5.78, 17.17) | 9.95(5.75, 17.23) | 104.43 | 3.31(2.55) | 9.93(6.3) |
| renal and urinary disorders | oedematous kidney | 12 | 322.45(180.21, 576.98) | 322(178.85, 579.72) | 3636.15 | 8.25(7.45) | 304.96(187.41) |
| renal and urinary disorders | renal vein thrombosis | 12 | 135.57(76.45, 240.4) | 135.38(76.68, 239.01) | 1563.79 | 7.05(6.25) | 132.28(81.91) |
| renal and urinary disorders | glomerulosclerosis | 12 | 125.01(70.53, 221.56) | 124.83(70.71, 220.38) | 1442.75 | 6.93(6.14) | 122.2(75.7) |
| renal and urinary disorders | renal tubular disorder | 12 | 27.25(15.45, 48.07) | 27.21(15.41, 48.04) | 301.6 | 4.76(3.97) | 27.09(16.85) |
| injury, poisoning and procedural complications | graft loss | 12 | 108.5(61.27, 192.14) | 108.35(61.37, 191.29) | 1252.64 | 6.73(5.94) | 106.36(65.93) |
| injury, poisoning and procedural complications | transplant failure | 12 | 33.96(19.25, 59.92) | 33.91(19.21, 59.87) | 381.06 | 5.08(4.29) | 33.72(20.97) |
| immune system disorders | intestine transplant rejection | 12 | 1169.58(628.51, 2176.44) | 1167.93(623.77, 2186.79) | 11626.48 | 9.92(9.07) | 970.7(577.3) |
| immune system disorders | pancreas transplant rejection | 12 | 445.19(247.36, 801.27) | 444.57(246.93, 800.4) | 4929.23 | 8.69(7.87) | 412.69(252.39) |
| vascular disorders | lymphocele | 12 | 112.02(63.24, 198.42) | 111.86(63.36, 197.48) | 1293.29 | 6.78(5.99) | 109.75(68.02) |
| blood and lymphatic system disorders | disseminated intravascular coagulation | 12 | 5.49(3.11, 9.67) | 5.48(3.1, 9.67) | 43.93 | 2.45(1.67) | 5.48(3.41) |
| infections and infestations | adenovirus infection | 11 | 28.17(15.57, 50.96) | 28.14(15.63, 50.66) | 286.5 | 4.81(3.99) | 28(17.05) |
| infections and infestations | bacterial infection | 11 | 4.19(2.32, 7.56) | 4.18(2.32, 7.53) | 26.61 | 2.06(1.25) | 4.18(2.55) |
| renal and urinary disorders | polyuria | 11 | 9(4.98, 16.26) | 8.99(4.99, 16.19) | 77.99 | 3.17(2.35) | 8.98(5.47) |
| investigations | transaminases increased | 11 | 3.32(1.83, 5.99) | 3.31(1.84, 5.96) | 17.75 | 1.73(0.91) | 3.31(2.02) |
| injury, poisoning and procedural complications | post procedural complication | 11 | 3.76(2.08, 6.79) | 3.75(2.08, 6.75) | 22.23 | 1.91(1.09) | 3.75(2.29) |
| blood and lymphatic system disorders | haemolytic anaemia | 11 | 8.16(4.51, 14.74) | 8.15(4.53, 14.67) | 68.87 | 3.02(2.21) | 8.14(4.96) |
| blood and lymphatic system disorders | coagulopathy | 11 | 4.32(2.39, 7.81) | 4.32(2.4, 7.78) | 28.05 | 2.11(1.29) | 4.32(2.63) |
| renal and urinary disorders | renal haemorrhage | 10 | 32.03(17.2, 59.65) | 31.99(17.09, 59.9) | 298.57 | 4.99(4.14) | 31.82(18.91) |
| investigations | cytomegalovirus test positive | 10 | 38.87(20.86, 72.42) | 38.83(20.74, 72.7) | 366.05 | 5.27(4.41) | 38.57(22.92) |
| investigations | immunosuppressant drug level increased | 10 | 22.23(11.94, 41.38) | 22.21(11.86, 41.59) | 201.74 | 4.47(3.61) | 22.12(13.15) |
| investigations | blood urine present | 10 | 3.45(1.86, 6.42) | 3.45(1.84, 6.46) | 17.41 | 1.79(0.93) | 3.45(2.05) |
| immune system disorders | graft versus host disease in skin | 10 | 32.32(17.35, 60.19) | 32.28(17.24, 60.44) | 301.41 | 5(4.15) | 32.1(19.08) |
| hepatobiliary disorders | hepatic necrosis | 10 | 16.72(8.98, 31.11) | 16.7(8.92, 31.27) | 147.16 | 4.06(3.2) | 16.65(9.9) |
| hepatobiliary disorders | cholestasis | 10 | 3.64(1.96, 6.78) | 3.64(1.94, 6.82) | 19.16 | 1.86(1.01) | 3.64(2.17) |
| respiratory, thoracic and mediastinal disorders | lung infiltration | 10 | 6.92(3.72, 12.88) | 6.92(3.7, 12.96) | 50.55 | 2.79(1.93) | 6.91(4.11) |
| infections and infestations | nocardiosis | 9 | 25.6(13.29, 49.29) | 25.57(13.39, 48.82) | 211.57 | 4.67(3.77) | 25.46(14.72) |
| infections and infestations | candida infection | 9 | 3.95(2.05, 7.59) | 3.94(2.06, 7.52) | 19.76 | 1.98(1.08) | 3.94(2.28) |
| renal and urinary disorders | renal haematoma | 9 | 115.74(59.81, 223.98) | 115.62(59.38, 225.14) | 1002.46 | 6.82(5.92) | 113.36(65.24) |
| immune system disorders | graft versus host disease in gastrointestinal tract | 9 | 30.54(15.86, 58.82) | 30.51(15.98, 58.26) | 255.52 | 4.92(4.03) | 30.35(17.54) |
| blood and lymphatic system disorders | haemolytic uraemic syndrome | 9 | 23.97(12.45, 46.15) | 23.95(12.54, 45.73) | 197.1 | 4.58(3.68) | 23.85(13.79) |
| respiratory, thoracic and mediastinal disorders | pneumothorax | 9 | 3.89(2.02, 7.47) | 3.88(2.03, 7.41) | 19.25 | 1.96(1.06) | 3.88(2.24) |
| metabolism and nutrition disorders | hyperuricaemia | 9 | 14.9(7.75, 28.68) | 14.89(7.8, 28.43) | 116.32 | 3.89(3) | 14.85(8.59) |
| infections and infestations | cytomegalovirus colitis | 8 | 27.9(13.92, 55.9) | 27.88(14.04, 55.36) | 206.3 | 4.79(3.85) | 27.75(15.51) |
| infections and infestations | escherichia infection | 8 | 6.96(3.48, 13.93) | 6.95(3.5, 13.8) | 40.74 | 2.8(1.85) | 6.95(3.89) |
| renal and urinary disorders | nephrosclerosis | 8 | 43.01(21.45, 86.26) | 42.97(21.64, 85.33) | 325.54 | 5.41(4.47) | 42.66(23.83) |
| renal and urinary disorders | azotaemia | 8 | 13.93(6.96, 27.88) | 13.92(7.01, 27.64) | 95.68 | 3.8(2.85) | 13.89(7.77) |
| investigations | blood creatinine decreased | 8 | 17.65(8.81, 35.34) | 17.63(8.88, 35.01) | 125.15 | 4.14(3.19) | 17.58(9.84) |
| investigations | low density lipoprotein increased | 8 | 7.41(3.7, 14.84) | 7.41(3.73, 14.71) | 44.29 | 2.89(1.94) | 7.4(4.14) |
| vascular disorders | arteritis | 8 | 85.15(42.35, 171.2) | 85.07(42.01, 172.27) | 654.97 | 6.39(5.44) | 83.84(46.74) |
| vascular disorders | haemodynamic instability | 8 | 7.66(3.83, 15.32) | 7.65(3.85, 15.19) | 46.19 | 2.93(1.99) | 7.64(4.28) |
| blood and lymphatic system disorders | splenomegaly | 8 | 4.54(2.27, 9.09) | 4.54(2.29, 9.02) | 22.07 | 2.18(1.24) | 4.54(2.54) |
| blood and lymphatic system disorders | lymphopenia | 8 | 3.92(1.96, 7.85) | 3.92(1.97, 7.78) | 17.4 | 1.97(1.03) | 3.92(2.19) |
| respiratory, thoracic and mediastinal disorders | acute pulmonary oedema | 8 | 9.35(4.67, 18.71) | 9.34(4.7, 18.55) | 59.51 | 3.22(2.28) | 9.33(5.22) |
| nervous system disorders | hemiplegia | 8 | 6.25(3.12, 12.5) | 6.24(3.14, 12.39) | 35.18 | 2.64(1.7) | 6.24(3.49) |
| metabolism and nutrition disorders | hypoalbuminaemia | 8 | 7.32(3.66, 14.64) | 7.31(3.68, 14.52) | 43.54 | 2.87(1.92) | 7.3(4.09) |
| infections and infestations | urinary tract infection enterococcal | 7 | 47(22.33, 98.91) | 46.96(22.3, 98.9) | 312.31 | 5.54(4.54) | 46.59(24.99) |
| infections and infestations | enterobacter infection | 7 | 29.05(13.82, 61.06) | 29.02(13.78, 61.12) | 188.45 | 4.85(3.85) | 28.88(15.51) |
| infections and infestations | mucormycosis | 7 | 19.37(9.22, 40.69) | 19.35(9.19, 40.75) | 121.43 | 4.27(3.27) | 19.29(10.37) |
| infections and infestations | escherichia urinary tract infection | 7 | 10.92(5.2, 22.92) | 10.91(5.18, 22.98) | 62.88 | 3.44(2.44) | 10.89(5.85) |
| infections and infestations | bronchopulmonary aspergillosis | 7 | 6.41(3.05, 13.46) | 6.41(3.04, 13.5) | 31.92 | 2.68(1.68) | 6.4(3.44) |
| infections and infestations | pyelonephritis | 7 | 5.58(2.66, 11.72) | 5.58(2.65, 11.75) | 26.29 | 2.48(1.48) | 5.57(3) |
| infections and infestations | wound infection | 7 | 5.22(2.49, 10.95) | 5.21(2.47, 10.97) | 23.82 | 2.38(1.38) | 5.21(2.8) |
| infections and infestations | urosepsis | 7 | 5.16(2.46, 10.83) | 5.16(2.45, 10.87) | 23.43 | 2.37(1.36) | 5.15(2.77) |
| infections and infestations | bacteraemia | 7 | 4.25(2.02, 8.91) | 4.24(2.01, 8.93) | 17.35 | 2.08(1.08) | 4.24(2.28) |
| renal and urinary disorders | renal cortical necrosis | 7 | 240.89(113.06, 513.29) | 240.7(112.07, 516.95) | 1603.69 | 7.85(6.83) | 231.05(122.69) |
| renal and urinary disorders | cystitis haemorrhagic | 7 | 12.18(5.8, 25.57) | 12.17(5.78, 25.63) | 71.62 | 3.6(2.6) | 12.15(6.53) |
| investigations | high density lipoprotein decreased | 7 | 13.5(6.43, 28.36) | 13.49(6.41, 28.41) | 80.79 | 3.75(2.75) | 13.46(7.24) |
| injury, poisoning and procedural complications | graft complication | 7 | 81.27(38.53, 171.42) | 81.2(38.56, 171.01) | 546.79 | 6.32(5.32) | 80.09(42.89) |
| injury, poisoning and procedural complications | renal transplant failure | 7 | 52.72(25.04, 111.01) | 52.68(25.01, 110.95) | 351.68 | 5.71(4.7) | 52.21(28) |
| injury, poisoning and procedural complications | wound complication | 7 | 13.74(6.54, 28.85) | 13.73(6.52, 28.92) | 82.42 | 3.78(2.77) | 13.7(7.36) |
| injury, poisoning and procedural complications | wound dehiscence | 7 | 13.54(6.45, 28.43) | 13.52(6.42, 28.47) | 81.01 | 3.75(2.75) | 13.5(7.25) |
| immune system disorders | lung transplant rejection | 7 | 73.81(35.01, 155.62) | 73.75(35.02, 155.32) | 496.01 | 6.19(5.18) | 72.83(39.02) |
| vascular disorders | ischaemia | 7 | 9.62(4.58, 20.19) | 9.61(4.56, 20.24) | 53.91 | 3.26(2.26) | 9.6(5.16) |
| hepatobiliary disorders | cholangitis | 7 | 8.38(3.99, 17.6) | 8.38(3.98, 17.65) | 45.41 | 3.06(2.06) | 8.37(4.5) |
| general disorders and administration site conditions | systemic inflammatory response syndrome | 7 | 12(5.71, 25.19) | 11.99(5.69, 25.25) | 70.36 | 3.58(2.58) | 11.97(6.43) |
| nervous system disorders | brain oedema | 7 | 3.73(1.78, 7.83) | 3.73(1.77, 7.86) | 13.97 | 1.9(0.9) | 3.73(2) |
| cardiac disorders | left ventricular hypertrophy | 7 | 10.58(5.04, 22.22) | 10.57(5.02, 22.26) | 60.56 | 3.4(2.4) | 10.55(5.67) |
| infections and infestations | human herpesvirus 8 infection | 6 | 85.77(38.29, 192.1) | 85.71(38.37, 191.44) | 494.93 | 6.4(5.32) | 84.46(43.01) |
| infections and infestations | fungaemia | 6 | 27.43(12.3, 61.19) | 27.41(12.27, 61.22) | 151.96 | 4.77(3.7) | 27.28(13.94) |
| infections and infestations | herpes simplex | 6 | 6.46(2.9, 14.39) | 6.45(2.89, 14.41) | 27.63 | 2.69(1.62) | 6.45(3.3) |
| renal and urinary disorders | renal arteriosclerosis | 6 | 175.91(78.05, 396.46) | 175.79(78.7, 392.64) | 1011.78 | 7.41(6.33) | 170.59(86.43) |
| renal and urinary disorders | nephropathy | 6 | 4.12(1.85, 9.18) | 4.12(1.84, 9.2) | 14.17 | 2.04(0.97) | 4.12(2.11) |
| investigations | immunosuppressant drug level decreased | 6 | 33.8(15.15, 75.44) | 33.78(15.12, 75.45) | 189.74 | 5.07(4) | 33.59(17.16) |
| investigations | bilirubin conjugated increased | 6 | 17.09(7.67, 38.1) | 17.08(7.65, 38.15) | 90.58 | 4.09(3.02) | 17.03(8.71) |
| investigations | urine output increased | 6 | 14.92(6.69, 33.25) | 14.91(6.68, 33.3) | 77.66 | 3.89(2.82) | 14.87(7.61) |
| investigations | blood uric acid increased | 6 | 7.2(3.23, 16.05) | 7.2(3.22, 16.08) | 32 | 2.85(1.78) | 7.19(3.68) |
| investigations | prothrombin time prolonged | 6 | 6.02(2.7, 13.41) | 6.02(2.7, 13.45) | 25.08 | 2.59(1.52) | 6.01(3.08) |
| immune system disorders | heart transplant rejection | 6 | 39.4(17.65, 87.97) | 39.38(17.63, 87.96) | 222.88 | 5.29(4.22) | 39.11(19.97) |
| vascular disorders | venous thrombosis | 6 | 10.01(4.49, 22.31) | 10.01(4.48, 22.36) | 48.56 | 3.32(2.25) | 9.99(5.11) |
| hepatobiliary disorders | hepatitis cholestatic | 6 | 6.98(3.13, 15.56) | 6.98(3.13, 15.59) | 30.7 | 2.8(1.73) | 6.97(3.57) |
| respiratory, thoracic and mediastinal disorders | non-cardiogenic pulmonary oedema | 6 | 48.15(21.55, 107.57) | 48.12(21.54, 107.48) | 274.55 | 5.58(4.5) | 47.73(24.36) |
| respiratory, thoracic and mediastinal disorders | atelectasis | 6 | 3.97(1.78, 8.84) | 3.97(1.78, 8.87) | 13.31 | 1.99(0.92) | 3.97(2.03) |
| gastrointestinal disorders | large intestine perforation | 6 | 5.82(2.61, 12.96) | 5.82(2.61, 13) | 23.91 | 2.54(1.47) | 5.81(2.97) |
| neoplasms benign, malignant and unspecified (incl cysts and polyps) | lymphoproliferative disorder | 6 | 14.4(6.46, 32.08) | 14.39(6.44, 32.14) | 74.55 | 3.84(2.77) | 14.35(7.34) |
| nervous system disorders | leukoencephalopathy | 6 | 10.56(4.74, 23.52) | 10.55(4.72, 23.56) | 51.77 | 3.4(2.33) | 10.53(5.39) |
| infections and infestations | cytomegalovirus gastrointestinal infection | 5 | 122.25(50.4, 296.52) | 122.18(50.58, 295.15) | 588.41 | 6.9(5.73) | 119.65(57.01) |
| infections and infestations | microsporidia infection | 5 | 111.78(46.12, 270.93) | 111.72(46.25, 269.88) | 538.17 | 6.78(5.61) | 109.61(52.26) |
| infections and infestations | pseudomonal bacteraemia | 5 | 45.17(18.73, 108.93) | 45.14(18.69, 109.05) | 214.15 | 5.49(4.33) | 44.8(21.45) |
| infections and infestations | viraemia | 5 | 27.52(11.43, 66.27) | 27.5(11.38, 66.43) | 127.08 | 4.77(3.62) | 27.38(13.12) |
| infections and infestations | jc virus infection | 5 | 23.06(9.58, 55.51) | 23.04(9.54, 55.66) | 105.01 | 4.52(3.36) | 22.95(11.01) |
| infections and infestations | brain abscess | 5 | 14.21(5.91, 34.2) | 14.21(5.88, 34.33) | 61.24 | 3.83(2.67) | 14.17(6.8) |
| infections and infestations | abdominal infection | 5 | 13.66(5.68, 32.86) | 13.65(5.65, 32.97) | 58.49 | 3.77(2.61) | 13.62(6.54) |
| infections and infestations | staphylococcal bacteraemia | 5 | 8.34(3.47, 20.06) | 8.34(3.45, 20.15) | 32.24 | 3.06(1.9) | 8.33(4) |
| infections and infestations | endocarditis | 5 | 6.65(2.76, 15.98) | 6.64(2.75, 16.04) | 23.94 | 2.73(1.57) | 6.64(3.19) |
| infections and infestations | staphylococcal sepsis | 5 | 6.48(2.69, 15.57) | 6.47(2.68, 15.63) | 23.12 | 2.69(1.54) | 6.47(3.1) |
| infections and infestations | hepatitis b | 5 | 5.39(2.24, 12.95) | 5.38(2.23, 13) | 17.83 | 2.43(1.27) | 5.38(2.58) |
| renal and urinary disorders | urinoma | 5 | 228(93.26, 557.45) | 227.87(92.5, 561.37) | 1086.27 | 7.78(6.6) | 219.21(103.75) |
| renal and urinary disorders | glomerulonephritis membranoproliferative | 5 | 67.76(28.05, 163.67) | 67.72(28.03, 163.59) | 324.83 | 6.06(4.9) | 66.94(32) |
| renal and urinary disorders | renal infarct | 5 | 23.24(9.65, 55.96) | 23.23(9.62, 56.12) | 105.94 | 4.53(3.37) | 23.14(11.09) |
| renal and urinary disorders | renal artery stenosis | 5 | 22.51(9.35, 54.2) | 22.5(9.31, 54.35) | 102.33 | 4.49(3.33) | 22.42(10.75) |
| investigations | urine analysis abnormal | 5 | 10.72(4.46, 25.79) | 10.72(4.44, 25.9) | 43.97 | 3.42(2.26) | 10.7(5.13) |
| injury, poisoning and procedural complications | complications of transplanted liver | 5 | 89.22(36.87, 215.87) | 89.17(36.91, 215.41) | 429.23 | 6.46(5.29) | 87.82(41.93) |
| injury, poisoning and procedural complications | postoperative wound complication | 5 | 25.67(10.66, 61.82) | 25.66(10.62, 61.99) | 117.96 | 4.68(3.52) | 25.55(12.25) |
| immune system disorders | acute graft versus host disease in skin | 5 | 13.29(5.52, 31.96) | 13.28(5.5, 32.08) | 56.65 | 3.73(2.57) | 13.25(6.36) |
| vascular disorders | arterial rupture | 5 | 42.75(17.73, 103.07) | 42.73(17.69, 103.22) | 202.24 | 5.41(4.25) | 42.42(20.31) |
| blood and lymphatic system disorders | aplasia pure red cell | 5 | 9.94(4.13, 23.9) | 9.93(4.11, 23.99) | 40.09 | 3.31(2.15) | 9.92(4.76) |
| general disorders and administration site conditions | tissue infiltration | 5 | 134.88(55.55, 327.46) | 134.8(55.8, 325.64) | 648.79 | 7.04(5.87) | 131.73(62.71) |
| general disorders and administration site conditions | necrosis | 5 | 5.87(2.44, 14.12) | 5.87(2.43, 14.18) | 20.18 | 2.55(1.4) | 5.86(2.81) |
| neoplasms benign, malignant and unspecified (incl cysts and polyps) | epstein-barr virus associated lymphoproliferative disorder | 5 | 20.73(8.61, 49.89) | 20.72(8.58, 50.05) | 93.48 | 4.37(3.21) | 20.64(9.9) |
| metabolism and nutrition disorders | hypovolaemia | 5 | 5.69(2.37, 13.68) | 5.69(2.36, 13.75) | 19.29 | 2.51(1.35) | 5.68(2.73) |
| skin and subcutaneous tissue disorders | skin necrosis | 5 | 6.27(2.61, 15.06) | 6.26(2.59, 15.12) | 22.09 | 2.65(1.49) | 6.26(3) |
| infections and infestations | cytomegalovirus gastritis | 4 | 138.43(51.34, 373.29) | 138.37(50.92, 375.98) | 532.67 | 7.08(5.79) | 135.14(58.93) |
| infections and infestations | parvovirus b19 infection | 4 | 49.1(18.35, 131.41) | 49.08(18.42, 130.77) | 186.81 | 5.61(4.33) | 48.67(21.36) |
| infections and infestations | enterococcal sepsis | 4 | 31.78(11.89, 84.94) | 31.77(11.92, 84.65) | 118.55 | 4.98(3.71) | 31.6(13.88) |
| infections and infestations | enterococcal bacteraemia | 4 | 28.13(10.53, 75.14) | 28.11(10.55, 74.9) | 104.09 | 4.81(3.54) | 27.98(12.3) |
| infections and infestations | epididymitis | 4 | 23.21(8.69, 61.98) | 23.2(8.71, 61.82) | 84.64 | 4.53(3.26) | 23.11(10.16) |
| infections and infestations | strongyloidiasis | 4 | 17.21(6.45, 45.94) | 17.21(6.46, 45.86) | 60.87 | 4.1(2.83) | 17.16(7.55) |
| infections and infestations | pneumonia klebsiella | 4 | 15.36(5.76, 40.99) | 15.35(5.76, 40.9) | 53.53 | 3.94(2.67) | 15.32(6.74) |
| infections and infestations | pneumonia cytomegaloviral | 4 | 14.87(5.57, 39.69) | 14.87(5.58, 39.62) | 51.6 | 3.89(2.62) | 14.83(6.52) |
| infections and infestations | human herpesvirus 6 infection | 4 | 13.85(5.19, 36.96) | 13.85(5.2, 36.9) | 47.56 | 3.79(2.52) | 13.81(6.08) |
| infections and infestations | pyelonephritis acute | 4 | 13.8(5.17, 36.83) | 13.8(5.18, 36.77) | 47.36 | 3.78(2.52) | 13.76(6.06) |
| infections and infestations | systemic candida | 4 | 12.2(4.57, 32.54) | 12.19(4.58, 32.48) | 41.01 | 3.61(2.34) | 12.17(5.35) |
| infections and infestations | hiv infection | 4 | 8.68(3.25, 23.15) | 8.68(3.26, 23.13) | 27.13 | 3.12(1.85) | 8.67(3.81) |
| infections and infestations | bacterial sepsis | 4 | 8.46(3.17, 22.56) | 8.45(3.17, 22.51) | 26.25 | 3.08(1.81) | 8.44(3.72) |
| infections and infestations | fungal skin infection | 4 | 6.97(2.62, 18.6) | 6.97(2.62, 18.57) | 20.43 | 2.8(1.53) | 6.96(3.06) |
| infections and infestations | abdominal abscess | 4 | 6.34(2.38, 16.89) | 6.33(2.38, 16.87) | 17.94 | 2.66(1.4) | 6.33(2.78) |
| infections and infestations | subcutaneous abscess | 4 | 5.5(2.06, 14.67) | 5.5(2.06, 14.65) | 14.72 | 2.46(1.19) | 5.5(2.42) |
| infections and infestations | aspergillus infection | 4 | 4.6(1.73, 12.28) | 4.6(1.73, 12.26) | 11.27 | 2.2(0.94) | 4.6(2.03) |
| renal and urinary disorders | renal artery thrombosis | 4 | 89.77(33.43, 241.06) | 89.72(33.67, 239.06) | 345.54 | 6.47(5.19) | 88.36(38.66) |
| renal and urinary disorders | renal atrophy | 4 | 20.78(7.78, 55.47) | 20.77(7.8, 55.34) | 74.99 | 4.37(3.1) | 20.7(9.1) |
| investigations | haptoglobin decreased | 4 | 34.15(12.77, 91.26) | 34.13(12.81, 90.94) | 127.88 | 5.08(3.82) | 33.93(14.91) |
| investigations | blood magnesium increased | 4 | 29.42(11.01, 78.61) | 29.41(11.04, 78.36) | 109.22 | 4.87(3.6) | 29.27(12.86) |
| investigations | antibody test positive | 4 | 14.99(5.62, 40) | 14.98(5.62, 39.91) | 52.07 | 3.9(2.63) | 14.95(6.57) |
| investigations | blood creatine increased | 4 | 6.07(2.28, 16.18) | 6.07(2.28, 16.17) | 16.9 | 2.6(1.33) | 6.06(2.67) |
| injury, poisoning and procedural complications | ureteric anastomosis complication | 4 | 1209.48(411.37, 3555.99) | 1208.91(411.36, 3552.74) | 3988.05 | 9.96(8.57) | 998.84(405.14) |
| injury, poisoning and procedural complications | kidney rupture | 4 | 145.44(53.91, 392.42) | 145.38(53.5, 395.03) | 559.37 | 7.15(5.86) | 141.81(61.81) |
| injury, poisoning and procedural complications | complications of transplant surgery | 4 | 49(18.31, 131.13) | 48.98(18.38, 130.51) | 186.39 | 5.6(4.33) | 48.57(21.31) |
| immune system disorders | acute allograft nephropathy | 4 | 1641.44(540.18, 4987.78) | 1640.66(536.82, 5014.32) | 5098.07 | 10.32(8.9) | 1276.29(503.59) |
| immune system disorders | chronic graft versus host disease | 4 | 7.79(2.92, 20.79) | 7.79(2.92, 20.76) | 23.65 | 2.96(1.69) | 7.78(3.42) |
| immune system disorders | acute graft versus host disease | 4 | 5.88(2.21, 15.69) | 5.88(2.21, 15.67) | 16.19 | 2.56(1.29) | 5.88(2.59) |
| blood and lymphatic system disorders | atypical haemolytic uraemic syndrome | 4 | 39.35(14.72, 105.22) | 39.33(14.76, 104.79) | 148.41 | 5.29(4.02) | 39.07(17.16) |
| hepatobiliary disorders | hepatic artery thrombosis | 4 | 96.15(35.78, 258.35) | 96.11(36.07, 256.08) | 370.27 | 6.56(5.29) | 94.54(41.35) |
| hepatobiliary disorders | bile duct stenosis | 4 | 22.82(8.55, 60.94) | 22.81(8.56, 60.78) | 83.09 | 4.51(3.24) | 22.72(9.99) |
| hepatobiliary disorders | hepatic fibrosis | 4 | 8.14(3.05, 21.71) | 8.14(3.06, 21.69) | 25 | 3.02(1.76) | 8.13(3.58) |
| gastrointestinal disorders | pancreatic pseudocyst | 4 | 33.84(12.66, 90.46) | 33.83(12.7, 90.14) | 126.69 | 5.07(3.8) | 33.64(14.78) |
| gastrointestinal disorders | intra-abdominal haemorrhage | 4 | 10.86(4.07, 28.98) | 10.86(4.08, 28.94) | 35.74 | 3.44(2.17) | 10.84(4.77) |
| gastrointestinal disorders | gastrointestinal necrosis | 4 | 9.18(3.44, 24.48) | 9.17(3.44, 24.43) | 29.08 | 3.2(1.93) | 9.16(4.03) |
| gastrointestinal disorders | ileus paralytic | 4 | 6.16(2.31, 16.44) | 6.16(2.31, 16.41) | 17.28 | 2.62(1.36) | 6.16(2.71) |
| gastrointestinal disorders | intestinal ischaemia | 4 | 4.96(1.86, 13.23) | 4.96(1.86, 13.22) | 12.64 | 2.31(1.04) | 4.96(2.18) |
| general disorders and administration site conditions | fibrosis | 4 | 8.68(3.26, 23.16) | 8.68(3.26, 23.13) | 27.14 | 3.12(1.85) | 8.67(3.82) |
| general disorders and administration site conditions | granuloma | 4 | 7.84(2.94, 20.91) | 7.84(2.94, 20.89) | 23.84 | 2.97(1.7) | 7.83(3.45) |
| neoplasms benign, malignant and unspecified (incl cysts and polyps) | renal cell carcinoma | 4 | 5.26(1.97, 14.02) | 5.26(1.97, 14.02) | 13.77 | 2.39(1.13) | 5.25(2.31) |
| cardiac disorders | ventricular hypertrophy | 4 | 9.35(3.51, 24.93) | 9.34(3.51, 24.89) | 29.76 | 3.22(1.96) | 9.33(4.11) |
| reproductive system and breast disorders | scrotal pain | 4 | 48.58(18.15, 130.01) | 48.56(18.23, 129.39) | 184.77 | 5.59(4.32) | 48.16(21.13) |
| infections and infestations | adenoviral haemorrhagic cystitis | 3 | 101.97(32.55, 319.41) | 101.93(32.7, 317.69) | 294.6 | 6.65(5.22) | 100.17(38.53) |
| infections and infestations | cerebral aspergillosis | 3 | 34.6(11.12, 107.68) | 34.59(11.1, 107.81) | 97.28 | 5.1(3.68) | 34.39(13.3) |
| infections and infestations | cytomegalovirus enterocolitis | 3 | 29.92(9.62, 93.06) | 29.91(9.6, 93.22) | 83.39 | 4.9(3.48) | 29.76(11.51) |
| infections and infestations | klebsiella sepsis | 3 | 18.35(5.91, 57.02) | 18.35(5.89, 57.19) | 49.05 | 4.19(2.78) | 18.29(7.08) |
| infections and infestations | stenotrophomonas infection | 3 | 16.65(5.36, 51.72) | 16.64(5.34, 51.86) | 43.99 | 4.05(2.64) | 16.6(6.43) |
| infections and infestations | gastroenteritis norovirus | 3 | 14.47(4.66, 44.94) | 14.46(4.64, 45.07) | 37.51 | 3.85(2.43) | 14.43(5.59) |
| infections and infestations | meningitis cryptococcal | 3 | 12.98(4.18, 40.29) | 12.97(4.16, 40.42) | 33.07 | 3.69(2.28) | 12.95(5.02) |
| infections and infestations | mycobacterial infection | 3 | 11.84(3.81, 36.77) | 11.84(3.8, 36.9) | 29.71 | 3.56(2.15) | 11.82(4.58) |
| infections and infestations | cytomegalovirus chorioretinitis | 3 | 11.23(3.62, 34.88) | 11.23(3.6, 35) | 27.9 | 3.49(2.07) | 11.21(4.34) |
| infections and infestations | wound infection staphylococcal | 3 | 10.68(3.44, 33.17) | 10.68(3.43, 33.29) | 26.27 | 3.41(2) | 10.66(4.13) |
| infections and infestations | urinary tract infection bacterial | 3 | 8.04(2.59, 24.95) | 8.03(2.58, 25.03) | 18.45 | 3(1.59) | 8.03(3.11) |
| infections and infestations | cytomegalovirus infection reactivation | 3 | 7.59(2.45, 23.57) | 7.59(2.44, 23.66) | 17.15 | 2.92(1.51) | 7.58(2.94) |
| infections and infestations | necrotising fasciitis | 3 | 6.07(1.95, 18.82) | 6.06(1.94, 18.89) | 12.67 | 2.6(1.18) | 6.06(2.35) |
| infections and infestations | oesophageal candidiasis | 3 | 5.4(1.74, 16.75) | 5.4(1.73, 16.83) | 10.74 | 2.43(1.02) | 5.39(2.09) |
| infections and infestations | clostridial infection | 3 | 5.27(1.7, 16.37) | 5.27(1.69, 16.43) | 10.38 | 2.4(0.98) | 5.27(2.04) |
| renal and urinary disorders | ureteral necrosis | 3 | 302.33(94.66, 965.58) | 302.23(95.09, 960.63) | 855.66 | 8.17(6.71) | 287.17(108.68) |
| renal and urinary disorders | urinary fistula | 3 | 167.31(53.07, 527.43) | 167.25(52.62, 531.6) | 481.74 | 7.34(5.9) | 162.55(62.19) |
| renal and urinary disorders | perinephric collection | 3 | 146.04(46.42, 459.43) | 145.99(46.84, 455.02) | 421.28 | 7.15(5.72) | 142.4(54.58) |
| renal and urinary disorders | kidney congestion | 3 | 143.61(45.66, 451.67) | 143.56(46.06, 447.44) | 414.34 | 7.13(5.69) | 140.08(53.7) |
| renal and urinary disorders | renal vessel disorder | 3 | 86.6(27.69, 270.85) | 86.57(27.78, 269.82) | 249.97 | 6.41(4.99) | 85.3(32.85) |
| renal and urinary disorders | renal vasculitis | 3 | 62.67(20.08, 195.54) | 62.64(20.1, 195.24) | 180.02 | 5.95(4.53) | 61.98(23.92) |
| renal and urinary disorders | urinary tract inflammation | 3 | 60.68(19.45, 189.31) | 60.66(19.46, 189.06) | 174.19 | 5.91(4.48) | 60.03(23.17) |
| renal and urinary disorders | bladder dilatation | 3 | 18.27(5.88, 56.78) | 18.27(5.86, 56.94) | 48.81 | 4.19(2.77) | 18.21(7.05) |
| renal and urinary disorders | ureteric obstruction | 3 | 14.38(4.63, 44.67) | 14.38(4.61, 44.82) | 37.26 | 3.84(2.43) | 14.35(5.56) |
| renal and urinary disorders | renal tubular acidosis | 3 | 10.02(3.23, 31.12) | 10.02(3.21, 31.23) | 24.32 | 3.32(1.91) | 10.01(3.88) |
| investigations | biopsy kidney abnormal | 3 | 92.16(29.45, 288.39) | 92.12(29.56, 287.12) | 266.13 | 6.5(5.07) | 90.68(34.91) |
| investigations | red blood cell schistocytes present | 3 | 57.83(18.54, 180.36) | 57.81(18.55, 180.18) | 165.81 | 5.84(4.42) | 57.24(22.1) |
| investigations | urinary casts | 3 | 50.83(16.31, 158.44) | 50.82(16.31, 158.39) | 145.23 | 5.65(4.23) | 50.38(19.46) |
| investigations | aspartate aminotransferase decreased | 3 | 40.45(12.99, 125.95) | 40.44(12.97, 126.04) | 114.58 | 5.33(3.91) | 40.16(15.53) |
| investigations | eosinophil count decreased | 3 | 12.44(4.01, 38.63) | 12.44(3.99, 38.77) | 31.49 | 3.63(2.22) | 12.41(4.81) |
| investigations | oxygen saturation abnormal | 3 | 8.2(2.64, 25.44) | 8.2(2.63, 25.56) | 18.93 | 3.03(1.62) | 8.19(3.17) |
| investigations | brain natriuretic peptide increased | 3 | 6.66(2.14, 20.66) | 6.65(2.13, 20.73) | 14.4 | 2.73(1.32) | 6.65(2.58) |
| investigations | protein total decreased | 3 | 5.29(1.71, 16.42) | 5.29(1.7, 16.49) | 10.43 | 2.4(0.99) | 5.29(2.05) |
| injury, poisoning and procedural complications | suture related complication | 3 | 29.26(9.41, 91) | 29.25(9.38, 91.17) | 81.43 | 4.86(3.44) | 29.1(11.26) |
| injury, poisoning and procedural complications | incisional hernia | 3 | 14.03(4.52, 43.58) | 14.03(4.5, 43.73) | 36.21 | 3.81(2.39) | 14(5.42) |
| injury, poisoning and procedural complications | gastrointestinal stoma complication | 3 | 13.27(4.27, 41.2) | 13.26(4.25, 41.33) | 33.93 | 3.73(2.31) | 13.23(5.13) |
| injury, poisoning and procedural complications | vascular pseudoaneurysm | 3 | 11.53(3.71, 35.79) | 11.52(3.7, 35.91) | 28.77 | 3.52(2.11) | 11.5(4.46) |
| injury, poisoning and procedural complications | post procedural haematoma | 3 | 11.12(3.58, 34.54) | 11.12(3.57, 34.66) | 27.58 | 3.47(2.06) | 11.1(4.3) |
| vascular disorders | lymphorrhoea | 3 | 141.25(44.92, 444.16) | 141.2(45.3, 440.09) | 407.61 | 7.11(5.67) | 137.84(52.85) |
| vascular disorders | endothelial dysfunction | 3 | 100.19(31.99, 313.79) | 100.16(32.14, 312.18) | 289.45 | 6.62(5.19) | 98.46(37.88) |
| vascular disorders | arteriovenous fistula | 3 | 40.08(12.87, 124.78) | 40.06(12.85, 124.86) | 113.47 | 5.31(3.89) | 39.79(15.38) |
| vascular disorders | extremity necrosis | 3 | 11.4(3.67, 35.41) | 11.4(3.66, 35.53) | 28.41 | 3.51(2.09) | 11.38(4.41) |
| vascular disorders | arterial thrombosis | 3 | 9.96(3.21, 30.9) | 9.95(3.19, 31.01) | 24.12 | 3.31(1.9) | 9.94(3.85) |
| vascular disorders | vasodilatation | 3 | 7.84(2.53, 24.33) | 7.84(2.52, 24.44) | 17.87 | 2.97(1.55) | 7.83(3.03) |
| blood and lymphatic system disorders | normochromic normocytic anaemia | 3 | 6.46(2.08, 20.05) | 6.46(2.07, 20.13) | 13.83 | 2.69(1.27) | 6.45(2.5) |
| hepatobiliary disorders | bile duct necrosis | 3 | 359.02(111.8, 1152.87) | 358.9(110.72, 1163.33) | 1007.71 | 8.4(6.93) | 337.84(127.28) |
| hepatobiliary disorders | hepatic infiltration eosinophilic | 3 | 302.33(94.66, 965.58) | 302.23(95.09, 960.63) | 855.66 | 8.17(6.71) | 287.17(108.68) |
| hepatobiliary disorders | portal vein thrombosis | 3 | 6.34(2.04, 19.66) | 6.33(2.03, 19.73) | 13.46 | 2.66(1.25) | 6.33(2.45) |
| gastrointestinal disorders | ileal perforation | 3 | 30.39(9.77, 94.54) | 30.38(9.75, 94.69) | 84.8 | 4.92(3.5) | 30.23(11.7) |
| gastrointestinal disorders | intestinal dilatation | 3 | 15.87(5.11, 49.29) | 15.86(5.09, 49.43) | 41.66 | 3.98(2.57) | 15.82(6.13) |
| gastrointestinal disorders | duodenal ulcer haemorrhage | 3 | 6.65(2.14, 20.63) | 6.65(2.13, 20.73) | 14.37 | 2.73(1.32) | 6.64(2.57) |
| general disorders and administration site conditions | hyperplasia | 3 | 21.3(6.85, 66.2) | 21.29(6.83, 66.36) | 57.81 | 4.41(2.99) | 21.22(8.22) |
| general disorders and administration site conditions | non-cardiac chest pain | 3 | 5.73(1.85, 17.78) | 5.73(1.84, 17.86) | 11.69 | 2.52(1.1) | 5.72(2.22) |
| neoplasms benign, malignant and unspecified (incl cysts and polyps) | adenocarcinoma | 3 | 8.64(2.78, 26.81) | 8.64(2.77, 26.93) | 20.22 | 3.11(1.69) | 8.62(3.34) |
| neoplasms benign, malignant and unspecified (incl cysts and polyps) | b-cell lymphoma | 3 | 5.72(1.84, 17.73) | 5.71(1.83, 17.8) | 11.66 | 2.51(1.1) | 5.71(2.21) |
| nervous system disorders | hypertensive encephalopathy | 3 | 32.33(10.39, 100.59) | 32.32(10.37, 100.73) | 90.55 | 5.01(3.59) | 32.15(12.44) |
| nervous system disorders | osmotic demyelination syndrome | 3 | 27.31(8.78, 84.92) | 27.3(8.76, 85.09) | 75.65 | 4.76(3.35) | 27.18(10.52) |
| nervous system disorders | white matter lesion | 3 | 17.42(5.61, 54.13) | 17.42(5.59, 54.29) | 46.29 | 4.12(2.7) | 17.37(6.73) |
| metabolism and nutrition disorders | hypoproteinaemia | 3 | 11.68(3.76, 36.27) | 11.68(3.75, 36.4) | 29.24 | 3.54(2.13) | 11.66(4.52) |
| cardiac disorders | mitral valve calcification | 3 | 30.45(9.79, 94.71) | 30.44(9.77, 94.87) | 84.96 | 4.92(3.5) | 30.28(11.72) |
| cardiac disorders | diastolic dysfunction | 3 | 6.44(2.08, 19.99) | 6.44(2.07, 20.07) | 13.77 | 2.69(1.27) | 6.43(2.49) |
| reproductive system and breast disorders | pelvic fluid collection | 3 | 30.29(9.74, 94.21) | 30.28(9.72, 94.38) | 84.48 | 4.91(3.49) | 30.12(11.66) |
| reproductive system and breast disorders | testicular swelling | 3 | 15.78(5.08, 49.02) | 15.78(5.06, 49.18) | 41.4 | 3.98(2.56) | 15.74(6.1) |
| musculoskeletal and connective tissue disorders | soft tissue necrosis | 3 | 30.45(9.79, 94.71) | 30.44(9.77, 94.87) | 84.96 | 4.92(3.5) | 30.28(11.72) |
| musculoskeletal and connective tissue disorders | muscle necrosis | 3 | 22.73(7.31, 70.66) | 22.73(7.29, 70.84) | 62.07 | 4.5(3.08) | 22.64(8.77) |
| eye disorders | blindness cortical | 3 | 41.43(13.3, 128.99) | 41.41(13.29, 129.07) | 117.46 | 5.36(3.94) | 41.12(15.9) |
